# Effect of vascular lesion preprocessing on Brain Intensity AbNormality Classification Algorithm (BIANCA) white matter hyperintensity segmentation

**DOI:** 10.1016/j.nicl.2026.104001

**Published:** 2026-05-11

**Authors:** Uchralt Temuulen, Ralf Mekle, Ivana Galinovic, Joachim E. Weber, Ulf Landmesser, Sebastian Kelle, Matthias Endres, Christian Stehning, Kersten Villringer, on behalf of the BeLOVE Study Group

**Affiliations:** a Center for Stroke Research Berlin, Charité-Universitätsmedizin Berlin, Corporate Member of Freie Universität Berlin and Humboldt-Universität zu Berlin, Berlin, Germany; b Berlin Institute of Health (BIH) at Charité-Universitätsmedizin Berlin, Berlin, Germany; c German Centre for Cardiovascular Research (DZHK), Partner site Berlin, Berlin, Germany; d Department of Neurology, Charité-Universitätsmedizin Berlin, Berlin, Germany; e Department of Cardiology, Charité-Universitätsmedizin Berlin, Berlin, Germany; f German Center for Neurodegenerative Diseases (DZNE), Partner site Berlin, Berlin, Germany; g German Center for Mental Health (DZPG), Partner site Berlin, Berlin, Germany; h Philips Clinical Science, Hamburg, Germany

**Keywords:** White matterhyperintensities, BIANCA, LOCATE, Automated segmentation, Stroke, Cerebral small vessel disease, FLAIR

## Abstract

**Background:**

White matter hyperintensity (WMH) segmentation using BIANCA (Brain Intensity AbNormality Classification Algorithm) in stroke populations is complicated by vascular lesions that share T2-hyperintense signal characteristics with WMH. Whether preprocessing decisions in the treatment of lesions affect segmentation accuracy has not yet been systematically evaluated in cerebrovascular cohorts involving multiple scanners.

**Methods:**

We compared fixed probability thresholds and locally adaptive thresholding with LOCATE (LOCally Adaptive Threshold Estimation), and three lesion-handling approaches: lesions present (non removed), replaced with zero intensities (removed), and replaced with normal-appearing white matter intensities (NAWM; inpainted), using the BeLOVE cohort (Berlin Longterm Observation of Vascular Events) and the WMH Segmentation Challenge dataset. Phase I (n = 89) optimized thresholding via stratified 5-fold cross-validation. Phase II assessed preprocessing effects on segmentation accuracy (Phase II-A, n = 89) and volume agreement (Phase II-B, n = 211).

**Results:**

BIANCA with LOCATE adaptive thresholding achieved moderate segmentation overlap (mean Dice 0.567), with lesion-level detection exceeding an F1 of 0.85. Preprocessing effects were statistically detectable but negligible in magnitude, with near-perfect agreement between all conditions. Stroke lesion volume was the highest-ranked predictor of volume differences between conditions; these scaled with lesion size but remained negligible in magnitude across all subgroups.

**Conclusions:**

BIANCA with LOCATE achieved moderate WMH segmentation performance with the best sensitivity-precision trade-off in this multi-scanner cerebrovascular cohort. Preprocessing effects were negligible at the group level. However, large lesions distort the FLAIR intensity distribution on which BIANCA relies for classification, which justifies lesion removal as a recommended preprocessing step.

## Highlights

• BIANCA/LOCATE adaptive thresholding balances detection and false positives.
• Zero-filling and white matter inpainting yield equivalent results.
• Lesion volume is the highest-ranked predictor of volume differences.
• Lesion removal reduces FLAIR intensity distortion caused by vascular lesions.

## 1 Introduction

In recent years white matter hyperintensities (WMH) have gained increased attention in the context of a better understanding of cerebral small vessel disease (cSVD). The term “white matter hyperintensity” has been used in the literature to refer broadly to T2-/FLAIR-hyperintense regions in the white matter reflecting a heterogeneous spectrum of pathologies, including gliosis, demyelination, axonal loss, and vascular changes (Fazekas et al., 1993, Gouw et al., 2011, Wardlaw et al., 2013). WMH distribution, pattern and severity have been acknowledged to be associated with several cerebrovascular risk factors (Lampe et al., 2019, Torres-Simon et al., 2024), cognitive decline, post-stroke depression (Ali et al., 2023), risk of stroke, and stroke outcome (Smith, 2010). However, they are also common in healthy elderly people (Wardlaw et al., 2013). Furthermore, periventricular WMHs tend to form confluent lesions alongside the ventricles, in contrast to deep (subcortical) WMH which appear more often as small punctuated lesions (Griffanti et al., 2018, Sundaresan et al., 2021). Manual segmentation, though regarded as the most precise method, is time consuming and subject to inter-rater variability (Mäntylä et al., 1997, Prins et al., 2004, van den Heuvel et al., 2006), especially when considering WMH segmentation in large cohort studies. Automated or semi-automated methods have therefore been developed to enable reproducible and efficient quantification (Anbeek et al., 2004, Admiraal-Behloul et al., 2005, Valdés Hernández et al., 2010, Caligiuri et al., 2015). Among these, BIANCA (Brain Intensity AbNormality Classification Algorithm; Griffanti et al., 2016) is a fully supervised k-nearest neighbour (k-NN) classifier integrated within FSL (https://fsl.fmrib.ox.ac.uk/fsl/) that uses T1-weighted and FLAIR intensities alongside spatial features for WMH segmentation. LOCATE (LOCally Adaptive Threshold Estimation; Sundaresan et al., 2019) extends BIANCA by replacing the global probability threshold with locally adaptive thresholds optimized for regional signal characteristics. Both tools are widely used in cerebrovascular research due to their transparency, established validation across cohorts, and integration within standard neuroimaging pipelines (Bordin et al., 2021, Ferris et al., 2023, Gaubert et al., 2023).

More recently, deep learning methods such as TrUE-Net (Sundaresan et al., 2022) and nnU-Net (Isensee et al., 2021) have demonstrated competitive or superior segmentation performance, particularly in multi-site settings where domain shift poses challenges for conventional classifiers. However, deep learning approaches require substantially larger training datasets, entail higher computational costs, and may lack interpretability regarding the influence of specific preprocessing decisions on segmentation output. Importantly, the fundamental challenge of intensity-based confounders in populations with focal vascular lesions — where vascular lesions share T2-hyperintense signal characteristics with WMH — is relevant to any automated segmentation approach, regardless of the underlying classification method.

Segmenting WMH in stroke patients is particularly challenging due to the presence of additional T2-hyperintense vascular lesions, including ischemic infarcts, lacunes, and intracranial hemorrhage, which may be misjudged by automated segmentation approaches (Bonkhoff et al., 2021, Schirmer et al., 2019). Recent evidence suggests that lesion-related segmentation errors can be globally distributed rather than focal, affecting the intensity histogram used for classification (King et al., 2020). Whether preprocessing decisions regarding vascular lesion handling — specifically whether lesions are removed from FLAIR images before algorithm training and testing — affect WMH segmentation accuracy has not been systematically evaluated. Furthermore, the extent to which MRI scanner type influences segmentation robustness relative to lesion-related factors in high vascular risk cohorts remains incompletely characterized.

Therefore, we aimed to assess:

1. The performance and robustness of BIANCA with LOCATE adaptive thresholding compared to fixed thresholds in a multi-scanner high vascular risk cohort
2. The effect of three preprocessing conditions (non removed, removed, inpainted) on WMH segmentation accuracy and volume estimates
3. Factors influencing WMH volume differences between preprocessing conditions, including lesion volume, scanner type, and lesion morphology.

## 2 Methods

### 2.1 Study design and datasets

We employed a two-phase validation framework for BIANCA (Griffanti et al., 2016) using the BeLOVE cohort (Berlin Longterm Observation of Vascular Events; https://drks.de/search/de/trial/DRKS00016852) and selected participants from the WMH Segmentation Challenge dataset (https://wmh.isi.uu.nl/). The study protocol was approved by the Ethics Committee of Charité-Universitätsmedizin Berlin (EA1/066/17). All BeLOVE participants provided written informed consent. Ethical approval for the Challenge dataset was obtained by the original data collectors.

**Fig. 1:**
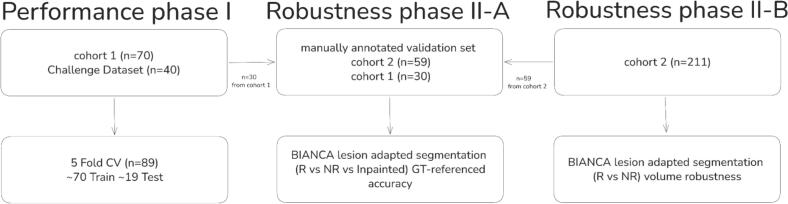
Flow chart of the multi-phase validation study design. Cohort 1 = BeLOVE subjects with ground-truth WMH masks (n = 70); Cohort 2 = independent BeLOVE validation participants (n = 211); Challenge Dataset = WMH Segmentation Challenge (Kuijf et al., 2019), after GE exclusion (n = 40). NR = non removed (lesions present), R = removed (lesion voxels zero-filled), Inpainted = vascular lesion voxels replaced with normal-appearing white matter (NAWM) intensity using FSL lesion_filling, GT = ground-truth manual WMH mask, CV = cross-validation. One participant from Cohort 2 was excluded due to failed preprocessing (fsl_anat), yielding n = 211 for Phase II-B.

Phase I served as parameter optimization, evaluating threshold strategies, preprocessing conditions, and cluster-based filtering using stratified 5-fold cross-validation with 10 predetermined seeds. Phase II applied the optimized configuration to assess preprocessing effects on segmentation accuracy (Phase II-A, n = 89) and volume-based agreement in an independent application sample (Phase II-B, n = 211; Fig. 1).

### 2.2 Datasets and scanner distribution

The combined dataset for Performance Phase I comprised n = 130 subjects from two sources. The BeLOVE cohort contributed n = 70 subjects (Cohort 1) scanned on three 3 T MRI systems: Siemens Prisma fit (n = 28, 40.0%), Siemens Tim Trio (n = 22, 31.4%), and Philips Ingenia (n = 20, 28.6%). All 70 subjects had ground-truth (manually delineated) WMH masks; 30 (42.9%) had concurrent vascular lesions requiring preprocessing, while 40 (57.1%) had WMH only. The WMH Segmentation Challenge dataset (Kuijf et al., 2019) contributed n = 60 subjects from three institutes: UMC Utrecht using a 3 T Philips Achieva (n = 20, 33.3%), NUHS Singapore using a 3 T Siemens TrioTim (n = 20, 33.3%), and VU Amsterdam using a 3 T GE Signa HDxt (n = 20, 33.3%). Each case included T1-weighted and FLAIR scans and manually delineated WMH masks. Scanner-specific acquisition parameters for all systems are provided in Supplemental Table S1. An independent BeLOVE validation cohort (Cohort 2, n = 212; n = 211 after exclusion of one subject due to failed preprocessing) contributed to both Phase II-A and Phase II-B. Of these, 59 subjects had ground-truth WMH masks and were included in Phase II-A alongside the 30 Cohort 1 subjects with concurrent vascular lesions (n = 89 total). Phase II-B used the full cohort (n = 211) for volume-based comparisons between preprocessing conditions.

### 2.3 Preprocessing pipeline

Brain extraction was performed using HD-BET (Isensee et al., 2019), which provides more reliable skull stripping in the presence of vascular lesions compared to the default FSL-BET implementation. Bias field correction of FLAIR images was performed separately using FAST (Zhang et al., 2001) with the −B option. T1-weighted image processing, FLAIR-to-T1 rigid-body registration, white matter mask generation, and ventricle distance map computation were performed using prepare_truenet (Sundaresan et al., 2022; https://github.com/v-sundaresan/truenet), which internally applies fsl_anat for T1 bias correction and tissue segmentation.

The white matter mask was generated within prepare_truenet using make_bianca_mask, which combines MNI-space white matter priors warped via nonlinear registration fields with CSF partial volume estimates from FAST tissue segmentation to constrain the mask to anatomically plausible white matter regions. This mask was applied during post-processing to eliminate extracerebral false positives. The ventricle distance map, derived from the lateral ventricle mask, was required for LOCATE's adaptive thresholding (*B + L;* Sundaresan et al., 2019). Visual quality control was performed at each processing stage.

#### 2.3.1 GE scanner exclusion

Given the documented sensitivity of BIANCA to acquisition parameter variations (Ferris et al., 2023), we assessed whether including the 20 GE Signa HDxt subjects from the Challenge dataset (VU Amsterdam) affected segmentation performance. We compared training with GE included (n = 60) versus excluded (n = 45, comprising Tim Trio, Philips, and Prisma fit) using stratified 5-fold cross-validation. All Cliff's Delta values were negligible (|δ| ≤ 0.047), confirming that GE inclusion did not meaningfully affect segmentation of non-GE subjects (Supplemental Table S2). However, GE test subjects (n = 5) showed medium-sized effects (*δ* = 0.147–0.36), likely reflecting differences in acquisition type rather than vendor identity per se. The GE Signa HDxt was the only system in our dataset using a 3D sagittal FLAIR acquisition (TR/TE/TI 8000/126/2340 ms, voxel size 0.98 × 0.98 × 1.20 mm^3^; Kuijf et al., 2019), whereas all other FLAIR data were acquired as 2D transversal sequences with slice thicknesses of 3 mm (Challenge data) or 5 mm (BeLOVE). We therefore excluded all 20 GE Signa subjects from subsequent analyses to ensure comparable acquisition geometry across the training set.

#### 2.3.2 Dataset composition after GE exclusion

After GE exclusion, the dataset comprised n = 110 subjects (70 BeLOVE + 40 Challenge). Of these, 21 subjects formed the LOCATE-only reserved subset (i.e., reserved exclusively for LOCATE threshold estimation and held out entirely from BIANCA cross-validation; 12 BeLOVE, 9 Challenge; 7 per scanner type). LOCATE was trained on n = 28 scanner-balanced subjects (7 per scanner type including GE) prior to GE exclusion; after exclusion, the 21 non-GE subjects were retained as the LOCATE pool for threshold estimation. The remaining n = 89 subjects constituted the BIANCA 5-fold cross-validation pool for Phase I and Phase II-A (58 BeLOVE + 31 Challenge).

### 2.4 Lesion delineation, removal, and inpainting

**Fig. 2:**
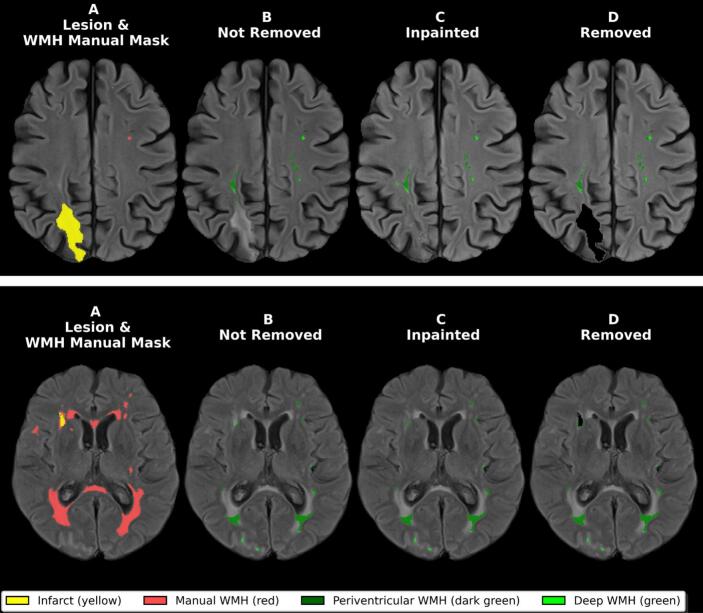
Representative WMH segmentation examples across preprocessing conditions. Representative axial FLAIR slices in two cases illustrating the operational distinction between focal vascular lesions and residual WMH, and the range of preprocessing effects. (A) Manual lesion and WMH delineation, illustrating the operational distinction applied throughout this study: focal vascular lesions (yellow) are explicitly delineated and removed prior to BIANCA segmentation, while residual FLAIR-hyperintense voxels are treated as the WMH segmentation target (red). (B) Non removed condition. (C) Inpainted condition. (D) Removed condition. Top row: well-segmented case showing minimal differences between non removed, inpainted, and removed conditions, with comparable WMH detection across all three preprocessing strategies. Bottom row: poorly-segmented case showing visible residual signal in the lesion-affected regions even in the removed condition, despite explicit lesion removal. This illustrates that preprocessing alone cannot fully prevent segmentation errors when extensive WMH burden coincides with focal vascular lesions. Color coding: infarct (yellow), manual WMH (red), periventricular WMH (dark green), deep WMH (green). (For interpretation of the references to colour in this figure legend, the reader is referred to the web version of this article.)

In population-based studies, WMH estimates inherently include small infarcts, lacunes, and other focal pathology, as these often share FLAIR signal characteristics with diffuse small vessel disease lesions and are difficult to separate by intensity alone. In the present study, we adopt a narrower operational distinction: focal vascular lesions (ischemic infarcts, lacunes, intracranial hemorrhage) are explicitly delineated and removed prior to BIANCA segmentation, while the remaining FLAIR-hyperintense voxels are treated as the WMH segmentation target. The goal of this preprocessing step is not to separate all pathological subtypes of white matter change, but to remove morphologically distinct focal lesions whose hyperintense FLAIR signal may confound intensity-based classification. This distinction is methodologically motivated: when WMH burden is used as a longitudinal biomarker, for example, to track therapy response), contamination by focal infarct volume, which can change abruptly during acute events and mask the slower developing, diffuse WMH changes that are intended to be detected by WMH segmentation. Fig. 2 (column A) illustrates the operational distinction between focal vascular lesions and the residual WMH treated as the segmentation target throughout our pipeline.

Lesions and WMH were manually delineated on FLAIR images using MRIcron (https://www.nitrc.org/projects/mricron). Vascular lesions were classified into five categories: (1) supratentorial ischemic infarcts; (2) infratentorial infarcts; (3) lacunes, defined as small (3–15 mm) subcortical cavitated lesions consistent with STRIVE criteria (Wardlaw et al., 2013); (4) mixed lesions (concurrent infarcts and lacunes); and (5) ICH.

Manual WMH masks for BeLOVE subjects were delineated, verified, and supervised by an experienced neuroradiologist (K.V., with 18 years of experience in stroke imaging). For Challenge subjects, WMH reference segmentations were obtained from Kuijf et al. (2019) and additionally reviewed and corrected by K.V.

We compared three preprocessing conditions: (1) Non removed: BIANCA segmentation performed on original FLAIR images, with vascular lesion voxels subtracted after segmentation from WMH maps; (2) Removed (zero-filled): vascular lesion voxel intensities replaced with zero values using fslmaths prior to BIANCA processing; and (3) Inpainted: vascular lesion voxels replaced with NAWM intensities using FSL's lesion_filling tool (Battaglini et al., 2012), which estimates surrounding tissue intensity to produce physiologically plausible signal distributions within the lesion cavity. Inpainting was included as an alternative that preserves the global intensity distribution while removing confounding hyperintense signal, addressing concerns that zero-filling may introduce artificial non-physiological intensity modes.

### 2.5 LOCATE adaptive thresholding

LOCATE (LOCally Adaptive Threshold Estimation; Sundaresan et al., 2019) was trained on 21 subjects (12 BeLOVE, 9 Challenge; scanner-balanced: 7 per scanner type), exceeding the recommended minimum of 20 training subjects. These subjects were entirely excluded from the BIANCA 5-fold cross-validation pool. LOCATE's internal leave-one-out procedure for generating unbiased lesion probability maps is a standard component of the algorithm and does not constitute data leakage, as it operates exclusively within the LOCATE training set.

### 2.6 Cross-validation strategy

We employed stratified 5-fold cross-validation with 10 predetermined seeds. Stratified folds ensure balanced representation of WMH severity levels and scanner types across all partitions, addressing potential scanner-specific information leakage in LOO-CV on mixed multi-scanner datasets. Repeated seeding (10 seeds) provides variance estimates, enabling estimation of performance variability across random partitions.

Subjects were stratified into three WMH severity groups (low, middle, high) based on ground-truth WMH volume (Ryu et al., 2014). Severity labels for Cohort 2 were adopted from the original dataset (Kuijf et al., 2019). For Cohort 1, severity was assigned using volume boundaries derived from Cohort 2 (low: ≤7.0 mL, middle: 7.0–27.4 mL, high: >27.4 mL). Stratified folds ensured balanced representation of these severity groups and scanner types (Tim Trio, Philips, Prisma fit) across all partitions. Per-subject performance metrics were computed as means across all 10 seeds × 5 folds (50 estimates per subject).

### 2.7 Phase I: Parameter optimization

#### 2.7.1 Threshold analysis

A systematic threshold analysis across the range 0.0–1.0 was performed on the inpainted condition to empirically determine the optimal voxel-level threshold. We compared three thresholding strategies: BIANCA with a fixed threshold of 0.85 (hereafter B0.85; Ferris et al., 2023), BIANCA with a fixed threshold of 0.90 (hereafter B0.90; Griffanti et al., 2016), and BIANCA with LOCATE adaptive thresholding (hereafter B + L; Sundaresan et al., 2019).

Conditions: training data inpainted, test data inpainted; n = 89 subjects; per-subject means across 10 seeds × 5-fold CV.

All three strategies were evaluated using inpainted images for both training and application (n = 89 subjects; per-subject means averaged across 10 predetermined seeds and 5-fold cross-validation).

Pairwise comparisons used Wilcoxon signed-rank tests with Bonferroni correction (k = 3 per metric, adjusted α = 0.0167).

#### 2.7.2 Cluster-based analysis

To evaluate lesion-level detection performance, we performed a cluster-based analysis complementing the voxel-level Dice coefficient. Optimal minimum cluster sizes were determined via grid search independently for each dataset: 16 voxels for BeLOVE and 8 voxels for Challenge subjects, reflecting differences in voxel resolution and WMH lesion characteristics between datasets. These dataset-specific thresholds were applied consistently across all subsequent analyses. Cluster-level metrics (lesion-level F1, precision, recall) were computed for each threshold configuration, assessing whether BIANCA detects individual WMH as spatially coherent clusters. Minimum cluster size filtering was applied exclusively to predicted masks; ground-truth masks were not filtered to avoid artificially inflating recall.

#### 2.7.3 Training condition comparison

To determine whether training data preprocessing affects segmentation, we compared all three conditions (Non removed, Removed, Inpainted) as training input, with the test condition fixed to inpainted and LOCATE adaptive thresholding. This design isolates the effect of training preprocessing on segmentation performance. For each pair, Wilcoxon signed-rank tests with Bonferroni correction (k = 3 per metric) and Cliff's Delta with bootstrapped 95% confidence intervals (Efron and Tibshirani, 1993) (1,000 iterations) were computed.

#### 2.7.4 Test condition comparison

Complementing the training comparison, we assessed test condition preprocessing effects by training exclusively on the inpainted condition and testing on all three conditions (Non removed, Removed, Inpainted), with LOCATE adaptive thresholding. Bland-Altman analyses quantified systematic agreement between conditions using non-parametric limits of agreement (2.5th/97.5th percentiles, median difference; Giavarina, 2015). Statistical testing followed the same procedure as the training comparison.

### 2.8 Phase II-A: preprocessing effect assessment (n = 89)

Based on Phase I results, Phase II employed a single BIANCA model trained on the inpainted condition (n = 59). Adaptive thresholding was performed using LOCATE, trained separately on a scanner-balanced reserved subset (n = 21).

Phase II-A assessed preprocessing effects in n = 89 subjects with ground-truth WMH masks. Unlike Phase I, which averaged across 10 seeds × 5-fold CV, Phase II-A applied the trained model once per subject, reflecting a real-world application scenario. Accuracy was evaluated using Dice coefficient, sensitivity, and precision across all three conditions (non removed, removed, inpainted), stratified by lesion type and scanner.

Scanner distribution:

Prisma fit n = 49 (55.1%), Tim Trio n = 29 (32.6%), Philips n = 11 (12.4%). Lesion types: lacunes n = 44 (49.4%), ischemic infarcts n = 29 (32.6%), mixed n = 10 (11.2%), infratentorial n = 5 (5.6%), ICH n = 1 (1.1%).

### 2.9 Phase II-B: volume-based agreement analysis (n = 211)

Phase II-B evaluated the generalizability of the optimized configuration by applying it to n = 211 BeLOVE participants not used for parameter optimization. As ground-truth WMH masks were unavailable for most subjects, this phase compared WMH volumes between non removed and removed conditions. Inclusion criteria: three months post-trigger event (TIA, heart failure, coronary artery disease, kidney failure, diabetes mellitus), age > 18 years, presence of vascular lesions, no MRI contraindications.

Scanner distribution: Prisma fit n = 124 (58.8%), Tim Trio n = 51 (24.2%), Philips n = 36 (17.1%). Lesion types: ischemic infarcts n = 96 (45.5%), lacunes n = 47 (22.3%), infratentorial n = 31 (14.7%), mixed n = 25 (11.8%), ICH n = 12 (5.7%).

Volume differences were assessed using Wilcoxon signed-rank tests with Cliff’s Delta, stratified by lesion type and scanner. SHAP predictor importance analysis identified predictors of volume discrepancies (lesion volume, scanner type, age, ARWMC score (Age-Related White Matter Changes; Wahlund et al., 2001), brain volume).

### 2.10 Statistical analysis

Statistical analyses were conducted in Python 3.12.3 using pandas 2.2 (McKinney, 2010), NumPy 2.0 (Harris et al., 2020), SciPy 1.13 (Virtanen et al., 2020), Matplotlib 3.9 (Hunter, 2007), seaborn 0.13.2 (Waskom, 2021), and scikit-learn 1.4.2 (Pedregosa et al., 2011). Continuous variables are reported as mean ± SD when normally distributed, and as median (interquartile range [IQR]) otherwise; categorical variables are presented as n (%).

For within-subject paired comparisons Wilcoxon signed-rank tests were used, for between-dataset comparisons (BeLOVE vs. Challenge) Mann-Whitney U tests. Effect sizes were quantified using Cliff's Delta (Cliff, 1993) with bootstrapped 95% confidence intervals (1000 iterations). Interpretation followed established benchmarks: |δ| < 0.147 negligible, 0.147–0.33 small, 0.33–0.474 medium, ≥0.474 large (Hess and Kromrey, 2004, Meissel and Yao, 2024).

Bland-Altman analysis assessed agreement between preprocessing conditions using non-parametric limits of agreement (2.5th/97.5th percentiles, median difference; Giavarina, 2015). To quantify agreement across the three preprocessing conditions, the Intraclass Correlation Coefficient (ICC, model 3,1: two-way mixed, single measures, consistency) was computed following the benchmarks of Koo and Li (2016), where values below 0.50 indicate poor, 0.50–0.75 moderate, 0.75–0.90 good, and above 0.90 excellent agreement. To provide positive evidence of equivalence rather than relying solely on the absence of significant differences, Two One-Sided Tests (TOST) were performed using paired Wilcoxon signed-rank tests with an equivalence margin of ±0.05 points on the metric scale (Schuirmann, 1987).

Spearman correlations assessed relationships between lesion volume and WMH volume differences. Trimmed estimates excluding the top 10% of lesion volumes (Wilcox, 2012, Pernet et al., 2012) with bootstrap 95% confidence intervals are reported alongside full untrimmed results for transparency.

A Random Forest model (500 trees, max depth = 10) was optimized via grid search with 5-fold cross-validation. Model performance is reported as out-of-bag R^2^ (an internal validation estimate based on samples not used during training of individual trees; Breiman, 2001) and Mean Absolute Error (MAE). Predictor importance was then quantified using SHAP (Lundberg and Lee, 2017, Molnar, 2022), computed exactly for the Random Forest via the TreeExplainer algorithm (Lundberg et al., 2020). Statistical significance threshold was set at α = 0.05 with Bonferroni correction applied per comparison family (Bland and Altman, 1995); specific family sizes are reported alongside each analysis and summarized in Supplemental Table S3.

## 3 Results

### 3.1 Population characteristics

The combined dataset after GE exclusion comprised n = 110 subjects (70 BeLOVE Cohort 1, 40 Challenge). After allocation of 21 subjects to the LOCATE training pool (scanner-balanced: 7 per scanner type), the remaining n = 89 subjects constituted the 5-fold cross-validation pool for Phase I. Scanner distribution: Tim Trio n = 35 (39.3%), Philips n = 33 (37.1%), Prisma fit n = 21 (23.6%). Median WMH volume was 21.45 mL (IQR 7.16–35.41). Subjects were stratified into three WMH severity groups based on ground-truth WMH volume cutoffs established from the Challenge dataset (n = 40): low (≤6.96 mL, n = 21), middle (6.96–27.40 mL, n = 32), and high (>27.40 mL, n = 36; Supplemental Table S4).

Phase II-A comprised n = 89 subjects with ground-truth WMH masks (n = 30 from Cohort 1, n = 59 from Cohort 2). Scanner distribution: Prisma fit n = 49 (55.1%), Tim Trio n = 29 (32.6%), Philips n = 11 (12.4%). Lesion types: lacunes n = 44 (49.4%), ischemic infarcts n = 29 (32.6%), mixed n = 10 (11.2%), infratentorial n = 5 (5.6%), ICH n = 1 (1.1%).

Phase II-B included n = 211 BeLOVE Cohort 2 participants (mean age 66.8 ± 11.5 years, 72.2% male). Scanner distribution: Prisma fit n = 124 (58.8%), Tim Trio n = 51 (24.2%), Philips n = 36 (17.1%). Lesion types: ischemic infarcts n = 96 (45.5%), lacunes n = 47 (22.3%), infratentorial infarcts n = 31 (14.7%), mixed n = 25 (11.8%), ICH n = 12 (5.7%). Median ARWMC score was 6.0 (IQR 3.0–10.0); median lesion volume 1.94 mL (IQR 0.69–8.26).

### 3.2 Phase I: parameter optimization (n = 89)

#### 3.2.1 Threshold analysis

Systematic threshold analysis across the range 0.0–1.0 on the inpainted condition identified the Dice optimum 0.83 (Dice 0.549, precision 0.644, sensitivity 0.544; Supplemental Fig. S1), confirming the threshold independently established by Ferris et al. (2023). To maintain comparability with published BIANCA validation studies, fixed-threshold analyses used 0.85.

Among the three thresholding strategies (training and test data both using the inpainted condition), BIANCA with LOCATE adaptive thresholding achieved the highest Dice coefficient (median 0.567) and sensitivity (0.687), while BIANCA with 0.85 and 0.90 fixed thresholds yielded higher precision (0.637 and 0.674) at the cost of lower sensitivity. Pairwise Wilcoxon signed-rank tests with Bonferroni correction (k = 3 per metric) showed that B + L significantly outperformed both fixed thresholds in Dice (Bonferroni-corrected *p* = 0.017 and 0.035), though effect sizes were negligible (*δ* = 0.077 and 0.097). Sensitivity differences were substantial (B + L vs B0.85: *δ* = 0.414, medium; B + L vs B0.90: *δ* = 0.523, large; both Bonferroni-corrected *p* < 0.001), while the precision disadvantage of B + L was small (δ =  − 0.213 and −0.300). (Supplemental Table S5).

Based on these results, LOCATE adaptive thresholding was selected for all subsequent analyses, as it achieved the optimal sensitivity–precision trade-off reflected in the highest Dice coefficient. Despite statistical significance, Dice effect sizes remained negligible (δ ≤ 0.10), indicating that the practical advantage of LOCATE over fixed thresholds was driven by its superior sensitivity. Performance was strongly severity-dependent (Supplemental Fig. S2), with high WMH burden subjects achieving Dice scores of 0.60–0.85, while low burden subjects clustered below 0.40.

#### 3.2.2 Cluster-based analysis

Cluster-level metrics complemented voxel-level evaluation across all thresholding strategies. Lesion-level F1 was high for all methods (B0.85: 0.88, B0.90: 0.86, B + L: 0.87), with cluster-level precision ranging from 0.88 to 0.93, confirming that detected clusters predominantly corresponded to true WMH lesions. Voxel-level Dice consistently underestimated detection performance: subjects with moderate Dice (0.2–0.5) frequently achieved cluster-level F1 above 0.80, particularly in low WMH burden cases (Supplemental Fig. S3). Optimal minimum cluster sizes were 16 voxels (BeLOVE) and 8 voxels (Challenge), determined via grid search and applied consistently across all subsequent analyses. Statistical comparisons revealed no meaningful differences in cluster-level F1 across thresholding strategies (all Cliff's δ ≤ 0.07, negligible; Supplemental Table S6). Cluster-level F1 showed less severity dependence than voxel-level Dice, with most subjects across all severity groups achieving F1 > 0.70 (Supplemental Fig. S4).

#### 3.2.3 Training condition comparison

Training condition had no meaningful effect on segmentation accuracy. When comparing all three training conditions (Non removed, Removed, Inpainted) with the test condition fixed to inpainted and LOCATE thresholding, mean Dice scores were virtually identical across conditions (0.567 ± 0.236; Supplemental Fig. S5). Pairwise Wilcoxon signed-rank tests with Bonferroni correction (k = 3 per metric, adjusted α = 0.0167) yielded no significant comparisons for Dice, sensitivity, or precision (smallest adjusted p = 0.104), and all Cliff's Delta values were negligible (max |δ| = 0.004, all 95% CIs encompassing zero; Supplemental Table S7).

Bland-Altman analysis confirmed tight agreement between training conditions, with 95% limits of agreement within ±0.006 for all metrics. The Removed versus Inpainted comparison showed consistently narrower limits of agreement than Non removed versus Removed, indicating that the filling strategy (zero-filling versus normal-appearing white matter inpainting) had even less influence than the presence or absence of lesions in training data. Three of nine uncorrected bias tests reached p < 0.05, but with negligible effect sizes (all Cliff's Delta 95% CIs encompassing zero), consistent with chance expectation.

Based on these results, inpainted was selected as the fixed training condition for the subsequent test condition comparison and all Phase II analyses.

#### 3.2.4 Test condition comparison

When training was fixed to the inpainted condition and all three test conditions were compared, test preprocessing produced statistically detectable but negligible differences. Mean Dice scores were identical across conditions (0.5670 ± 0.2363; Supplemental Fig. S6). Pairwise comparisons with Bonferroni correction (k = 3 per metric, adjusted α = 0.0167) showed no significant differences for Dice, while sensitivity and precision reached significance in 5 of 6 comparisons (adjusted p < 0.01). However, all Cliff's Delta values were negligible (max |δ| = 0.003, well below the |δ| ≥ 0.147 threshold; Supplemental Table S8). The opposing directional shifts — sensitivity slightly higher after removal (BIANCA detects previously obscured WMH) and precision slightly higher without removal — cancelled out in the Dice coefficient.

Bland-Altman analysis confirmed near-perfect agreement, with 95% LoA within ± 0.002 across all metrics and maximum bias of 0.0002. These LoA were approximately threefold narrower than those observed in the training condition comparison). The systematic bias pattern is consistent with the mechanistic expectation that removing hyperintense lesions normalizes the intensity distribution, enabling detection of subtle WMH that were previously obscured.

### 3.3 Phase II-A: preprocessing effect assessment (n = 89)

Phase II-A applied the optimized model (trained on inpainted, LOCATE thresholding, scanner-balanced training set n = 59) to 89 subjects with ground-truth WMH masks (n = 30 from Cohort 1, n = 59 from Cohort 2; mean age 68.9 ± 10.5 years, 73.0% male) in a single-run application scenario. Mean Dice scores were virtually identical across conditions (non removed: 0.576 ± 0.263, removed: 0.575 ± 0.263, inpainted: 0.575 ± 0.263).

Wilcoxon signed-rank tests with Bonferroni correction (k = 3 per metric, adjusted α = 0.0167) revealed opposing directional shifts: both removal conditions produced marginally higher sensitivity than Non removed (δ =  − 0.024 and − 0.022), while Non removed yielded marginally higher precision (*δ* = 0.012 and 0.013). All Cliff's Delta values were negligible (max |δ| = 0.024). Removed and Inpainted did not differ significantly for any metric (all adjusted p ≥ 0.095). Intraclass Correlation Coefficient (3,1) exceeded 0.999 for all metrics (Koo and Li, 2016), confirming that more than 99.9% of variance was attributable to between-subject differences. Two One-Sided Tests (TOST) equivalence testing (margin ±0.05) provided positive evidence of equivalence for all nine comparisons (all p < 0.001).

**Fig. 3:**
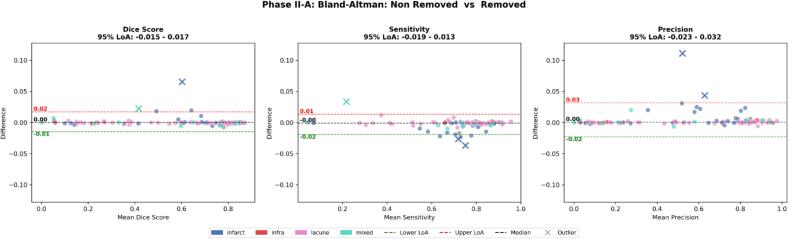
Bland-Altman agreement analysis for non removed versus removed conditions (Phase II-A, n = 89). Bland-Altman plots assess agreement between non removed and removed conditions across Dice coefficient, sensitivity, and precision. 95% limits of agreement (LoA) were narrow across all metrics (Dice: [−0.015, 0.017]; sensitivity: [−0.019, 0.013]; precision: [−0.023, 0.032]), with most points clustering near zero difference. Dashed lines indicate median difference (black), upper LoA (red), and lower LoA (green). Crosses denote outliers (beyond mean ± 2.5 SD). Color coding indicates lesion types. (For interpretation of the references to colour in this figure legend, the reader is referred to the web version of this article.)

Bland-Altman analysis revealed 95% LoA of [−0.015, 0.017] for Dice (non removed vs removed), narrowing approximately 10-fold for the removed versus inpainted comparison ([−0.002, 0.002]; Fig. 3).

**Fig. 4:**
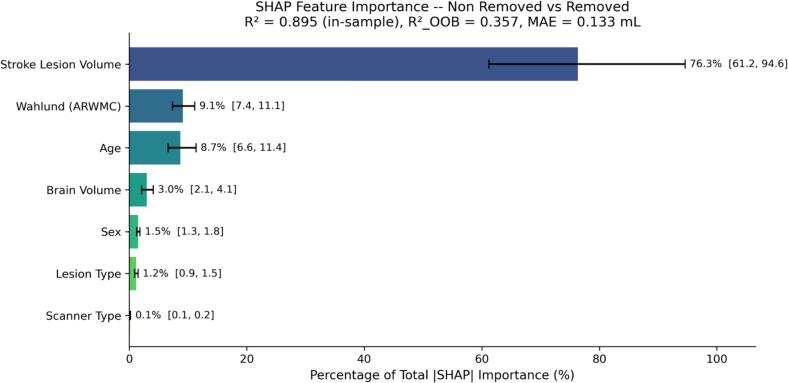
SHAP predictor importance for WMH volume differences between non removed and removed conditions (Phase II-A, n = 89). Random Forest model (explanatory R^2^ = 0.895, internally validated R^2^ = 0.357, MAE = 0.133 mL). Bars represent percentage of total mean |SHAP| importance with bootstrap 95% CIs (1000 iterations). Lesion volume was the highest-ranked predictor (76.3%). ARWMC = Age-Related White Matter Changes score (Wahlund et al., 2001).

SHAP predictor importance analysis identified lesion volume as the highest-ranked predictor of WMH volume differences between non removed and removed conditions (76.3%, 95% CI [61.2, 94.6]; explanatory R^2^ = 0.895, internally validated R^2^ = 0.357, MAE = 0.133 mL), followed by ARWMC score (Wahlund et al., 2001; 9.1%) and age (8.7%). Scanner type contributed 0.1%. Nearly identical importance profiles were observed for non removed versus inpainted (lesion volume 77.9%, 95% CI [61.7, 96.9]; explanatory R^2^ = 0.904, internally validated R^2^ = 0.386, MAE = 0.130 mL). The discrepancy between explanatory and internally validated R^2^ reflects that this Random Forest model was used to explain which factors drive volume differences, not to predict differences in new data; predictor importance rankings remained stable across both comparisons, confirming that the filling strategy does not alter which factors drive volume differences (Fig. 4).

### 3.4 Phase II-B: Volume-based agreement (n = 211)

Phase II-B applied the optimized configuration to 211 independent BeLOVE participants without ground-truth WMH masks. Based on the Phase II-A convergence analysis confirming equivalence between removed and inpainted conditions (all ICC > 0.999), this phase compared WMH volumes between non removed and removed conditions only.

**Volume differences by lesion type.** Ischemic infarcts (n = 96) and mixed lesions (n = 25) showed significant volume differences after Bonferroni correction (k = 15, adjusted α = 0.0033) across total, periventricular, and deep WMH compartments (Table 1; all p < 0.001 for infarcts; mixed: total p < 0.001, periventricular p = 0.003, deep p = 0.004). Removed consistently yielded higher WMH volumes. Lacunes showed marginal significance for periventricular WMH only (padj = 0.018). Infratentorial lesions and ICH were non-significant (all padj ≥ 0.47). Median absolute differences ranged from 0.04–0.14 mL for infarcts, 0.07–0.29 mL for mixed lesions, and 0.01–0.06 mL for lacunes. All Cliff’s Delta values were negligible (max |δ|=0.056 for ICH periventricular, n = 12; max |δ|=0.048 for mixed; max |δ|=0.033 for infarcts).

**Table 1:** Comparison of WMH segmentation volumes between non removed and removed conditions (Phase II-B, n = 211).

WMH	NR (mL) (median, IQR)	R (mL) (median, IQR)	Diff (mL) (median, IQR)	Higher	p (Bonf)	Cliff's Delta [95% CI]	Effect size
infratentorial strokes n=31							
Total	10.2 (7.14-20.02)	10.24 (7.12-20.11)	0.05 (0.02-0.1)	Removed	1.000 ns	0.00 [-0.2685, 0.2884]	negligible
Peri	7.99 (5.46-15.4)	7.92 (5.49-15.41)	0.04 (0.02-0.06)	Non Removed	1.000 ns	-0.00 [-0.2737, 0.2863]	negligible
Deep	1.23 (0.99-3.02)	1.23 (0.99-3.11)	0.01 (0.0-0.03)	Removed	1.000 ns	0.00 [-0.2883, 0.2852]	negligible

lacunes n=47							
Total	20.71 (12.98-31.08)	20.67 (12.99-31.4)	0.06 (0.02-0.15)	Non Removed	0.071 ns	-0.01 [-0.2413, 0.2142]	negligible
Peri	15.64 (9.65-21.94)	15.75 (9.62-22.15)	0.05 (0.01-0.09)	Removed	0.018 *	-0.01 [-0.2413, 0.2187]	negligible
Deep	3.31 (1.47-6.06)	3.31 (1.46-6.05)	0.02 (0.01-0.06)	Removed	0.477 ns	-0.01 [-0.2441, 0.23]	negligible

infarcts n=96							
Total	17.02 (11.19-24.39)	17.49 (11.43-25.49)	0.14 (0.05-0.61)	Removed	<0.001 ***	-0.03 [-0.1961, 0.1207]	negligible
Peri	12.82 (7.55-16.85)	13.1 (7.53-17.49)	0.1 (0.04-0.35)	Removed	<0.001 ***	-0.03 [-0.1991, 0.1271]	negligible
Deep	2.81 (1.49-5.42)	2.89 (1.51-5.78)	0.04 (0.02-0.15)	Removed	<0.001 ***	-0.03 [-0.1941, 0.1244]	negligible

mixed (infarcts+lacunes) n=25							
Total	21.46 (12.68-29.94)	21.66 (12.8-31.06)	0.29 (0.14-0.64)	Removed	<0.001 ***	-0.05 [-0.376, 0.2769]	negligible
Peri	15.58 (9.49-20.93)	15.79 (9.68-20.76)	0.17 (0.07-0.34)	Removed	0.003 **	-0.05 [-0.3633, 0.2704]	negligible
Deep	4.66 (1.38-6.69)	4.63 (1.36-7.37)	0.07 (0.03-0.19)	Non Removed	0.004 **	-0.05 [-0.36, 0.2737]	negligible

intracranial hemorrhage n=12							
Total	16.17 (12.79-17.42)	16.16 (12.62-19.33)	0.19 (0.09-0.78)	Non Removed	1.000 ns	-0.03 [-0.5139, 0.4306]	negligible
Peri	11.2 (8.21-13.91)	11.95 (8.19-14.19)	0.11 (0.07-0.34)	Removed	1.000 ns	-0.06 [-0.5417, 0.4028]	negligible
Deep	2.8 (1.61-3.84)	2.86 (1.59-3.81)	0.07 (0.02-0.33)	Removed	1.000 ns	0.01 [-0.4724, 0.4861]	negligible

Bonferroni correction applied per family (k = 15 tests, 5 lesion types × 3 compartments, corrected α = 0.0033). IQR = interquartile range, mL = millilitre, Diff = median absolute difference, Total = total WMH volume, Peri = periventricular WMH, Deep = deep WMH. Effect sizes (Cliff's Delta) with bootstrap 95% CIs (1000 iterations); thresholds: negligible |δ|<0.147, small 0.147–0.33, medium 0.33–0.474, large ≥0.474 (Romano et al., 2006).

**Volume differences by scanner.** All scanner types showed significant differences (Bonferroni-corrected, k = 9, 3 scanner types × 3 compartments, adjusted α = 0.0056; Supplemental Table S9), with Philips (n = 36) showing the largest median absolute differences (total 0.18 mL, δ =  − 0.06) compared to Prisma fit (n = 124, total 0.11 mL, δ =  − 0.02) and Tim_Trio (n = 51, total 0.09 mL, δ =  − 0.02). However, Philips subjects also had higher baseline WMH volumes (median 24.12 mL vs 14.67–15.58 mL for Siemens), so absolute differences may partly reflect the size-dependent scaling observed in the lesion-type analysis. All effect sizes remained negligible (max |δ|=0.06).

**SHAP predictor importance.** Lesion volume remained the highest-ranked predictor (64.8%, R^2^ = 0.930), followed by ARWMC score (12.4%; Wahlund et al., 2001) and scanner type (9.8%; Supplemental Fig. S7). Compared to Phase II-A (lesion volume 76.3%, scanner type 0.1%), scanner type increased from 0.1% to 9.8%, becoming the third-ranked predictor, consistent with the improved Philips representation in Phase II-B (17.0% vs 12.4% in Phase II-A). Full SHAP importance profiles are shown in Fig. 4.

Spearman rank correlations between lesion volume and WMH volume difference (removed minus non removed) were computed for each lesion type and WMH compartment (Supplemental Table S10). Ischemic infarcts (n = 96) showed the strongest and most consistent correlations across all three compartments (trimmed: total ρ = 0.62, 95% CI [0.44, 0.77]; deep ρ = 0.62, 95% CI [0.45, 0.75]; periventricular ρ = 0.61, 95% CI [0.41, 0.77]; all Bonferroni-corrected *p* < 0.001), confirming the size-dependent scaling of preprocessing effects observed in Phase II-A.

Lacunes (n = 47) showed moderate correlations that reached significance in the full sample (total ρ = 0.59, Bonferroni-corrected *p* < 0.001; periventricular ρ = 0.57, Bonferroni-corrected *p* < 0.001) but were attenuated in the trimmed analysis (total ρ = 0.45, Bonferroni-corrected *p* = 0.04; periventricular ρ = 0.44, Bonferroni-corrected *p* = 0.05), suggesting that the correlation is partly driven by subjects with the largest lesion volumes. Deep WMH correlations were weaker for lacunes (trimmed ρ = 0.29, Bonferroni-corrected *p* = 0.92).

Mixed lesions (n = 25), infratentorial lesions (n = 31), and intracranial hemorrhage (n = 12) showed no significant correlations after Bonferroni correction (all Bonferroni-corrected *p* ≥ 0.05). Confidence intervals for these subgroups were wide, reflecting limited statistical power, and results should be interpreted with caution.

## 4 Discussion

This study evaluated the robustness of BIANCA for automated WMH segmentation across three preprocessing conditions (non removed, removed, inpainted) in a multi-scanner high vascular risk cohort, revealing three principal findings. First, BIANCA with LOCATE adaptive thresholding achieved a mean Dice coefficient of 0.567 across stratified cross-validation, with cluster-level F1 exceeding 0.85 across all thresholding strategies, confirming robust detection of individual WMH even where voxel-level overlap was modest. Second, preprocessing condition produced statistically detectable but negligible effects on all segmentation metrics (all Cliff's Delta values <0.025, ICC > 0.999), with zero-filling and NAWM-based inpainting yielding equivalent results (Bland–Altman 95% LoA within ± 0.007). Third, lesion volume emerged as the highest-ranked predictor of WMH volume differences between preprocessing conditions (SHAP importance 64.8–76.3%), with size-dependent scaling confirmed by Spearman correlations. Despite all statistically significant effects (p < 0.001), no effect reached a magnitude warranting concern for group-level analyses (max |δ| = 0.040), supporting lesion removal as a recommended preprocessing step based on methodological consistency rather than bias magnitude.

### 4.1 Segmentation performance in context

BIANCA with LOCATE adaptive thresholding achieved a mean Dice coefficient of 0.567 (SD 0.236) across the Phase I cohort (n = 89), with pronounced dependence on WMH burden: high-burden subjects achieved high Dice scores while low-burden subjects clustered well below. This overall performance falls within the range reported across BIANCA validation studies (0.52–0.85; Griffanti et al., 2016, Ling et al., 2018, Bordin et al., 2021), with variability reflecting differences in dataset characteristics, WMH burden, training sample size (Wulms et al., 2022), and lesion morphology. The wide dispersion is driven primarily by the low WMH burden group, where small WMH volumes inherently produce low Dice scores due to the metric's sensitivity to small denominators. Cluster-level metrics provide a more informative assessment for low-burden cases: lesion-level F1 exceeded 0.85 across all thresholding strategies, confirming that BIANCA detects the majority of individual WMH as spatially coherent clusters even when voxel-level overlap is modest. This dissociation between voxel-level Dice and cluster-level detection has been previously reported in WMH segmentation (Kuijf et al., 2019) and underscores the importance of evaluating both metric types when assessing segmentation performance in heterogeneous cohorts.

LOCATE achieved the highest Dice coefficient (0.567), followed by B0.85 (0.545) and B0.90 (0.540), though the advantage over B0.85 was negligible in magnitude (*δ* = 0.077). The methods differed primarily in sensitivity and precision balance: LOCATE showed a medium-to-large sensitivity advantage (*δ* = 0.41–0.52) at the cost of a small precision reduction (δ =  − 0.21 to − 0.30). This contrasts with Ferris et al. (2023), who found a fixed 0.85 threshold superior to LOCATE in chronic stroke patients (Dice 0.60). The discrepancy likely reflects differences in cohort composition and validation design. Ferris et al. (2023) used a smaller single-site cohort with leave-one-out cross-validation, whereas our stratified 5-fold design drew from a substantially larger and more scanner-heterogeneous training pool. Larger and more diverse training sets may better capture the variability in WMH appearance that LOCATE requires for reliable local threshold estimation, potentially explaining why LOCATE performed comparably to a fixed threshold in the smaller, more homogeneous Ferris cohort but showed advantages in our multi-scanner dataset. Moreover, LOCATE was developed and validated predominantly in aging and cerebrovascular populations without large focal stroke lesions (Sundaresan et al., 2019), and its spatial priors may be partially violated in populations where hyperintense stroke lesions alter local intensity distributions.

### 4.2 Preprocessing effects: systematic but negligible

The central finding of this study is that preprocessing condition (Non removed, Removed, Inpainted) produces statistically detectable but negligible effects on WMH segmentation. All Cliff's Delta values across both phases remained below 0.025 (well below the |δ| ≥ 0.147 threshold for a meaningful effect), Intraclass Correlation Coefficient (3,1) exceeded 0.999, and Two One-Sided Tests equivalence testing confirmed formal equivalence within ± 0.05 points for all metrics. The statistical significance observed for sensitivity and precision reflects the high power of the paired design (89–211 subjects) rather than a meaningful difference in segmentation output.

We therefore frame these findings proportionately: preprocessing condition is a detectable but negligible source of variation in BIANCA WMH segmentation. While lesion removal represents a recommended preprocessing step for methodological consistency, the effect magnitude does not warrant characterization as a bias requiring correction, nor does it justify concerns about comparability between studies using different preprocessing approaches at the group level. The opposing directional shifts in sensitivity (removed > non removed) and precision (non removed > removed) cancel in the Dice coefficient, consistent with the mechanistic expectation that removing hyperintense lesions reduces distortion of the FLAIR intensity distribution, enabling detection of previously obscured subtle WMH alongside some additional false positives.

From a methodological perspective, these opposing shifts carry asymmetric consequences for downstream analyses. Systematic underestimation of WMH burden, as observed in the non removed condition, attenuates associations between WMH volume and outcome variables (e.g., cognitive decline, recurrence) through regression dilution (Hutcheon et al., 2010). Such attenuation bias reduces statistical power and may obscure true brain–behavior relationships in epidemiological analyses. In contrast, the modest overestimation introduced by additional false positives in the removed condition distributes approximately uniformly across subjects, preserving between-subject rank ordering and thus maintaining the validity of group-level correlations. While neither effect reached a magnitude of practical significance in our data (max |δ| = 0.024), the directional asymmetry provides an additional methodological rationale for preferring lesion removal: it avoids the type of systematic measurement error most likely to bias downstream inference.

Further, that lesion removal reduces FLAIR intensity distortion is empirically supported: FLAIR intensity distributions showed significantly higher skewness in the non removed condition compared to removed (mean difference + 0.028, Wilcoxon p < 0.001, n = 209), with 91.2% of subjects showing positive skewness shifts. The magnitude of distortion scaled with lesion volume (Spearman ρ = 0.84, p < 0.001), with large lesions (≥5 mL) producing approximately 67-fold greater skewness shifts than small lesions (<1 mL). Kurtosis was not significantly affected (p = 0.574), confirming that the distortion is specific to the upper intensity tail rather than a global distributional shift (Supplemental Fig. S8).

The convergence analysis (removed vs inpainted) provides direct evidence that zero-filling and NAWM-based inpainting produce equivalent results (Bland–Altman 95% LoA within ± 0.007, no significant bias after Bonferroni correction), isolating lesion removal per se as the active preprocessing step. This addresses the concern that zero-filling may introduce artificial non-physiological intensity distributions: neither filling strategy meaningfully differs from the other.

### 4.3 Lesion volume as highest-ranked predictor

SHAP analysis consistently identified lesion volume as the highest-ranked predictor of WMH volume differences between preprocessing conditions across both phases. Size-dependent scaling was confirmed by Spearman correlations in the full Phase II-B cohort, with the strongest associations observed for ischemic infarcts and lacunes (full statistics in Supplemental Table S10). This is consistent with the mechanistic explanation that larger lesions cause greater distortion of the FLAIR intensity distribution, affecting BIANCA's classification of subtle WMH (King et al., 2020, González-Villà et al., 2017, Gelineau-Morel et al., 2012). However, all effect sizes remained negligible (max |δ| = 0.040), confirming that the size-dependent pattern does not translate to a magnitude warranting concern for group-level analyses. The moderate performance of the this Random Forest model is consistent with its explanatory rather than predictive purpose and reflects the inherent variability in volume differences at the subject level when using a small set of features.

### 4.4 Scanner effects: acquisition parameters and sample composition

Scanner type emerged as the second-ranked SHAP predictor in Phase II-B (9.8%) but contributed less than 0.1% in Phase II-A. This increase is consistent with the improved Philips representation (17.0% vs 12.4%), confirming that studies with limited scanner diversity may systematically underestimate scanner-specific effects. The pattern is not explained by training–test mismatch, as Philips constituted approximately 33% of the training data: rather, the larger Philips validation sample in Phase II-B provides sufficient statistical power to detect scanner-specific volume differences that were obscured in the smaller Phase II-A sample. When stratified by scanner, Philips showed approximately 2-fold larger median volume differences (0.18 mL) compared to Siemens scanners (0.09–0.11 mL). However, Philips subjects also had higher baseline WMH volumes, so absolute differences may partly reflect size-dependent scaling rather than scanner-specific effects per se. All scanner-stratified effect sizes remained negligible (max |δ|=0.06). These findings confirm that k-NN classifiers like BIANCA are sensitive to acquisition parameter variations (Ferris et al., 2023), though k-NN approaches have been shown to outperform other automated methods across different scanner vendors (Heinen et al., 2019). The absolute magnitude of scanner effects does not reach a level that would compromise group-level comparisons when scanner-balanced training data are used.

A closer examination of the acquisition parameters (Supplemental Table S11) suggests that the GE-specific effect observed during cross-validation likely reflects acquisition parameters rather than scanner brand. The GE Signa HDxt was the only scanner in our dataset using a 3D sagittal FLAIR acquisition with 1.2 mm slice thickness, whereas all retained FLAIR data were acquired as 2D transversal sequences with 3–5 mm slice thickness. 3D versus 2D FLAIR acquisitions differ in contrast characteristics and partial volume effects, both of which can alter the intensity distribution on which BIANCA's k-NN classifier relies. At the opposite end of the resolution gradient, the BeLOVE acquisitions used 5 mm slices, which is at the upper end of clinically used FLAIR protocols. Increased partial volume averaging at this resolution likely reduces sensitivity to small or thin periventricular lesions and is consistent with the lower Dice values observed in low-burden cases (Section 4.1). The three acquisition regimes in our data therefore span a wide geometry gradient, from 1.2 mm isotropic 3D FLAIR (GE), through 3 mm 2D FLAIR (Challenge cohort), to 5 mm 2D FLAIR (BeLOVE), and segmentation performance tracks this gradient more closely than scanner brand. Within the retained 2D FLAIR acquisitions, TR ranged from 8000 to 11,000 ms and TE from 82 to 125 ms across vendors, yet the residual scanner effect remained negligible, suggesting that BIANCA tolerates moderate TR/TE variation when scanner-balanced training is used. Slice thickness and acquisition dimensionality (3D vs. 2D) therefore appear to be more plausible drivers of the GE effect than TR/TE differences or vendor identity. However, because the GE system was the only acquisition combining a 3D readout with 1.2 mm slices, the contribution of dimensionality (3D vs. 2D) cannot be statistically separated from that of slice thickness within our dataset; both are plausible drivers and likely act jointly. This pattern suggests that harmonization strategies should prioritize matching acquisition geometry over vendor matching.

### 4.5 Comparison with deep learning approaches

BIANCA remains widely used for WMH segmentation in cerebrovascular research due to its transparency, established validation, and integration within the FSL ecosystem. Deep learning methods such as TrUE-Net (Sundaresan et al., 2022) and nnU-Net (Isensee et al., 2021) have demonstrated competitive or superior performance in multi-site settings, with TrUE-Net showing particular robustness to domain shift. However, these methods require substantially larger training datasets and computational resources, may lack interpretability for understanding preprocessing effects, and their sensitivity to vascular lesion confounders has not been systematically evaluated. Our findings regarding preprocessing effects and scanner sensitivity are likely relevant to any intensity-based segmentation approach, whether k-NN or deep learning, as the underlying mechanism (FLAIR intensity histogram distortion by hyperintense lesions) operates independently of the classification method. Future studies should evaluate whether deep learning approaches show comparable, reduced, or amplified sensitivity to preprocessing decisions in vascular populations.

### 4.6 Limitations

Several limitations warrant consideration. First, while our validation cohorts were substantial (Phase I n = 89, Phase II-B n = 211), small-to-moderate sized lesions predominated; further validation with larger lesions and greater numbers of intracerebral hemorrhage would provide additional insight. Second, only Siemens and Philips scanners were included after GE exclusion (Section 2.2.1), limiting generalizability to 2D FLAIR acquisitions with slice thicknesses of 3–5 mm. The GE exclusion was empirically justified (medium-sized Cliff's Delta for GE test subjects) and likely reflects acquisition geometry, namely the fact that the GE Signa HDxt was the only 3D sagittal FLAIR acquisition with 1.2 mm slice thickness, rather than vendor identity per se. Future studies should evaluate BIANCA with dedicated training data for 3D FLAIR acquisitions to determine whether the observed effect is specific to acquisition geometry or vendor-specific intensity characteristics. Third, this single-center study cannot fully assess variability introduced by different imaging protocols or operator-dependent factors in true multi-site settings. Fourth, FLAIR bias correction was performed using FAST rather than N4 (Tustison et al., 2010); while FAST performs well on FLAIR images, interactions between vendor-specific on-scanner corrections and post-processing bias correction may influence the performance of intensity-based segmentation methods. Fifth, while BeLOVE WMH masks were delineated and verified by a single neuroradiologist (K.V.), formal inter-rater reliability metrics were not obtained. Sixth, the male predominance (72.2% in Phase II-B) limits generalizability to female populations, though sex showed negligible SHAP importance (0.2%) across all analyses. Seventh, while increased partial volume averaging at 5 mm slice thickness may reduce sensitivity to small or thin periventricular lesions, the absence of 1 mm 2D FLAIR data in our cohort precludes direct quantification of the slice-thickness contribution to overall segmentation performance. The lower Dice values observed in low-burden cases are consistent with the well-established sensitivity of the Dice metric to small denominators (Kuijf et al., 2019), but we cannot determine whether thinner slices would have improved performance generally, or only in specific subgroups.

### 4.7 Implications and future directions

Our findings support a practical recommendation to apply lesion removal (either zero-filling or NAWM-based inpainting) prior to BIANCA WMH segmentation in cerebrovascular cohorts. This recommendation is based on the mechanistic finding that lesion removal reduces FLAIR intensity distortion.

For practical implementation, we recommend lesion removal before BIANCA training and testing using either zero-filling or inpainting, LOCATE adaptive thresholding as default for multi-scanner cohorts, and scanner-balanced training data that includes all scanner types present in the target cohort. Future investigations should explore whether deep learning approaches show reduced sensitivity to preprocessing decisions, evaluate longitudinal stability of preprocessing effects as lesions evolve over time, and assess cross-manufacturer generalizability with dedicated training data for underrepresented scanner families. In the current study, vascular lesion masks were manually delineated, which limits full automation of the preprocessing pipeline. Future work should evaluate whether automated stroke lesion segmentation tools can provide sufficiently accurate lesion masks to enable fully automated lesion removal prior to WMH segmentation.

## 5 Conclusion

This study evaluated how lesion preprocessing affects automated WMH segmentation using BIANCA in a multi-scanner high vascular risk cohort. Across three preprocessing conditions (non removed, removed, inpainted), segmentation accuracy remained practically unchanged. Zero-filling and inpainting produced equivalent results, confirming that lesion removal itself is the relevant preprocessing step, regardless of filling strategy. Lesion volume was the strongest predictor of volume differences between conditions. We recommend lesion removal prior to WMH segmentation, as it reduces FLAIR intensity distortion caused by vascular lesions. Future studies should investigate generalizability to multi-site settings and deep learning-based segmentation methods.

### 5.1 The BeLOVE study group

Ahmadi M, Boldt LH, Buchmann N, Eckardt KU, Edelmann F, Endres M, Gerhardt H, Grittner U, Hübner N, Heege J, Landmesser U, Mai K, Müller DN, Nolte CH, Pinto RM, Piper SK, Pischon T, Rattan S, Rohrpasser-Napierkowski I, Schönrath K, Schulz-Menger J, Schweizerhof O, Weber JE.

## Declaration of Generative AI and AI-assisted technologies in the writing process

During the preparation of this work the author used OpenAI’s ChatGPT-4 and Anthropic’s Claude to assist in writing and correcting the analysis code, and to support manuscript revision. These tools were used to enhance code efficiency, troubleshoot errors, and improve language clarity. After using these tools/services, the author reviewed and edited the content as needed and takes full responsibility for the content of the publication.

## CRediT authorship contribution statement

**Uchralt Temuulen:** Writing – review & editing, Writing – original draft, Visualization, Validation, Resources, Methodology, Investigation, Formal analysis, Data curation, Conceptualization. **Ralf Mekle:** Writing – review & editing, Formal analysis. **Ivana Galinovic:** Writing – review & editing, Conceptualization. **Joachim E. Weber:** Writing – review & editing, Conceptualization. **Ulf Landmesser:** Writing – review & editing. **Sebastian Kelle:** Writing – review & editing. **Matthias Endres:** Writing – review & editing. **Christian Stehning:** Writing – review & editing. **Kersten Villringer:** Writing – review & editing, Writing – original draft, Supervision, Project administration, Conceptualization.

## Funding

M.E. received funding from the Deutsche Forschungsgemeinschaft (DFG) under Germany's Excellence Strategy (EXC-2049 – 390688087), the Collaborative Research Center ReTune (TRR 295 – 424778381), the Clinical Research Group KFO 5023 BeCAUSE-Y (project 2, EN343/16–1), the Research Group FOR 5930 AbsInVasc (project 6, EN343/18–1), as well as from Bundesministerium für Bildung und Forschung (BMBF), Deutsches Zentrum für Neurodegenerative Erkrankungen (DZNE), Deutsches Zentrum für Herz-Kreislauf-Forschung (DZHK), Deutsches Zentrum für Psychische Gesundheit (DZPG), the European Union, the Corona Foundation, and Fondation Leducq.

S.K. received funding from the DZHK, partner site Berlin, and Deutsche Forschungsgemeinschaft (DFG)—SFB 1470.

U.L. received funding by the Friede Springer Foundation.

## Declaration of competing interest

The authors declare the following potential conflicts of interest with respect to the research, authorship, and/or publication of this article: U.T., K.V., R.M., I.G. declare that they have no known competing financial interest or personal relationships that could have appeared to have influenced the work reported in this manuscript. M.E. reports grants from Bayer and Ipsen, and fees for lectures and/or consulting paid to Charité from Amgen, AstraZeneca, Bayer Healthcare, BMS, and Daiichi Sankyo, all outside the submitted work. M.E. holds the following unpaid leadership and fiduciary roles: Board of Directors, European Academy of Neurology; Member, German Society of Neurology; Member, International Society of Cerebral Blood Flow & Metabolism; Member, American Heart Association/American Stroke Association; Member, World Stroke Organization; Fellow, European Stroke Organisation. M.E. also holds a paid personal contract with the German Center for Neurodegenerative Diseases (DZNE). U.L. has received speaker or advisory honoraria from Abbott, Boston Scientific, Amgen, Bayer, Novo Nordisk, Pfizer, and Sanofi. S.K. reports grants from Philips Healthcare, BioVentrix, Berlin-Chemie, MSD/Bayer, Novartis, AstraZeneca, Siemens, and Myocardial Solutions, and serves on the advisory board for MSD/Bayer, BioVentrix, and Myocardial Solutions. C.S. is an employee of Philips Healthcare.

## Data Availability

Analysis scripts for WMH segmentation, preprocessing, statistical testing and figure generation are available at https://github.com/mendeltem/Bianca_Evaluation under the MIT License. Source imaging data cannot be shared publicly due to participant privacy restrictions under the European General Data Protection Regulation (GDPR). Processed, de-identified derivative data can be obtained from the corresponding author upon reasonable request and completion of appropriate data sharing agreements with the Center for Stroke Research Berlin.
