# Supplementary material for: Effect of vascular lesion preprocessing on Brain Intensity AbNormality Classification Algorithm (BIANCA) white matter hyperintensity segmentation
Source: Neuroimage Clin. 2026 May 11;50:104001. doi: 10.1016/j.nicl.2026.104001 (PMC13195355; doi:10.1016/j.nicl.2026.104001)
Supplement: Supplementary Data 1 [file mmc1.docx]

supplemental-material.docs:

**Supplemental Table S1.**

|  | **BeLOVE** | | | **WMH Challenge** | |
| --- | --- | --- | --- | --- | --- |
| **Parameter** | **SiemensTim Trio** | **SiemensPrisma fit** | **PhilipsIngenia** | **PhilipsAchieva**  **(Utrecht)** | **SiemensTrioTim**  **(Singapore)** |
| **FLAIR** | | | | | |
| **Sequence type** | 2D FLAIR | 2D T2_tse_dark_fluid | 2D FLAIR_SPIR | 2D FLAIR | 2D FLAIR |
| **TR (ms)** | 8000 | 9000 | 9000 | 11,000 | 9000 |
| **TE (ms)** | 96 | 83 | 120 | 125 | 82 |
| **TI (ms)** | 2370 | 2500 | 2500 | 2800 | 2500 |
| **Voxel size (mm³)** | — | — | — | 0.96×0.95×3.00 | 1.00×1.00×3.00 |
| **Matrix** | 256×232 | 320×224 | 232×228 | — | — |
| **Slice thickness (mm)** | 5 | 5 | 5 | 3 | 3 |
| **T1-weighted** | | | | | |
| **Sequence type** | 3D T1_MPRAGE | 3D T1_MPRAGE | 3D sT1W_3D_TFE | 3D T1w | 3D T1w |
| **TR (ms)** | 2300 | 2200 | 3000 | 7.9 | 2300 |
| **TE (ms)** | 3.0 | 2.32 | 2.94 | 4.5 | 1.9 |
| **TI (ms)** | — | — | — | — | 900 |
| **Voxel size (mm³)** | 1.00×1.00×1.00 | 0.9×0.9375×0.9375 | 0.55×0.3906×0.3906 | 1.00×1.00×1.00 | 1.00×1.00×1.00 |
| **Slice thickness (mm)** | 1 | 0.9 | 1 | 1 | 1 |
| **Flip angle (α)** | 9° | 8° | 8° | — | — |

*Scanner-specific acquisition parameters for FLAIR and T1-weighted sequences across all MRI systems used in this study. All scanners operated at 3T field strength. BeLOVE parameters were obtained from scanner protocols; Challenge parameters are from Kuijf et al. (2019). Dashes indicate parameters not available from the respective source.*

**Supplemental Table S2.** Scanner-stratified impact of GE scanner inclusion in the BIANCA training set on segmentation performance (Phase I).


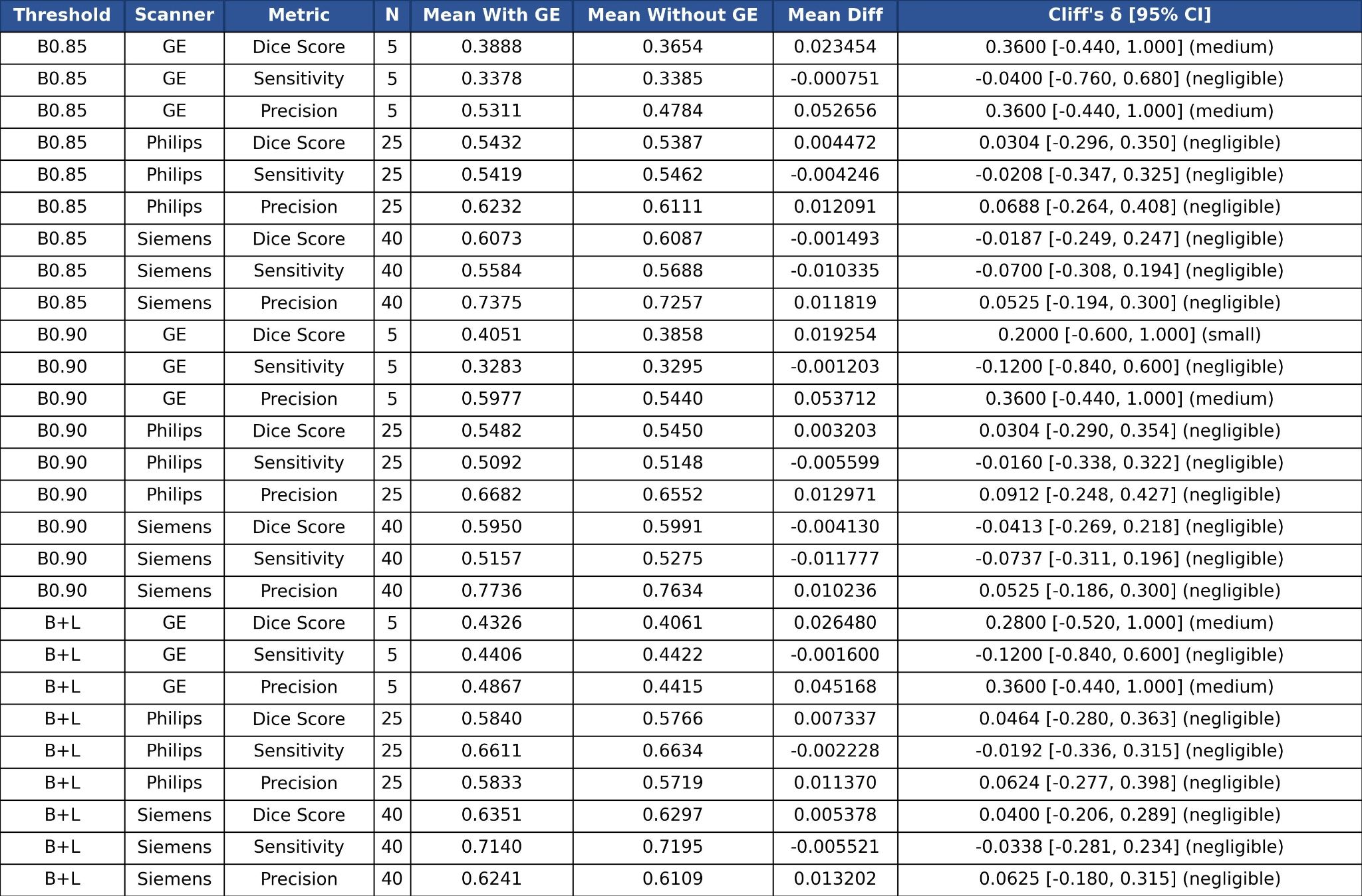


Performance metrics (Dice score, sensitivity, precision) were compared between models trained with GE data included (n=60 training subjects) and models trained without GE data (n=45 training subjects) for each scanner type separately. The "With GE" and "Without GE" training sets follow a nested design, where the Without GE set is a strict subset of the With GE set. Mean Diff = mean difference (With GE minus Without GE). Effect sizes are reported as Cliff's Delta (δ) with bootstrapped 95% confidence intervals (1,000 iterations, seed=42). Effect sizes were classified as negligible (|δ| < 0.147), small (0.147 ≤ |δ| < 0.33), medium (0.33 ≤ |δ| < 0.474), or large (|δ| ≥ 0.474) following Hess and Kromrey (2004). N = number of test subjects per scanner group. GE test subjects (n=5) showed medium-sized effects, but this subgroup is too small for reliable inference. Philips (n=25) and Siemens (n=40) subgroups showed negligible effects throughout.

**Supplemental Table S3.** Bonferroni correction family structure.


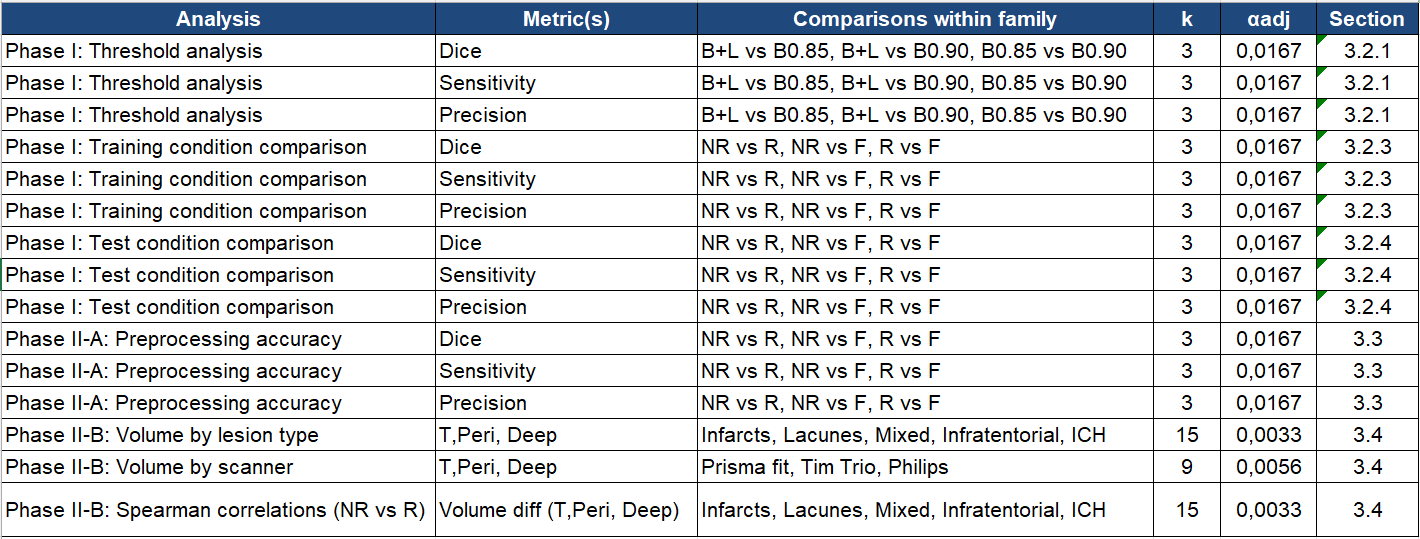


*Each row defines one comparison family with the number of tests (k) and the adjusted significance threshold (αadj = 0.05/k). Families were defined per metric within each analysis to control the family-wise error rate. NR = Non removed; R = removed; F = filled (inpainted); B+L = BIANCA + LOCATE; B0.85 = BIANCA with 0.85 threshold; B0.90 = BIANCA with 0.90 threshold; T = total WMH volume; Peri = periventricular; Deep = deep WMH. Phase I families correct across pairwise comparisons within each metric. Phase II-A families correct across preprocessing conditions within each metric. Phase II-B families correct across the full combination of subgroups × WMH compartments (Total, periventricular, deep) within each stratification variable; k reflects the number of comparisons × three compartments. All Bonferroni corrections are conservative; Cliff's Delta effect sizes and 95% bootstrap confidence intervals are reported alongside p-values throughout.*

***Supplemental Table S4*** WMH severity group definitions and volume distributions across study phases.


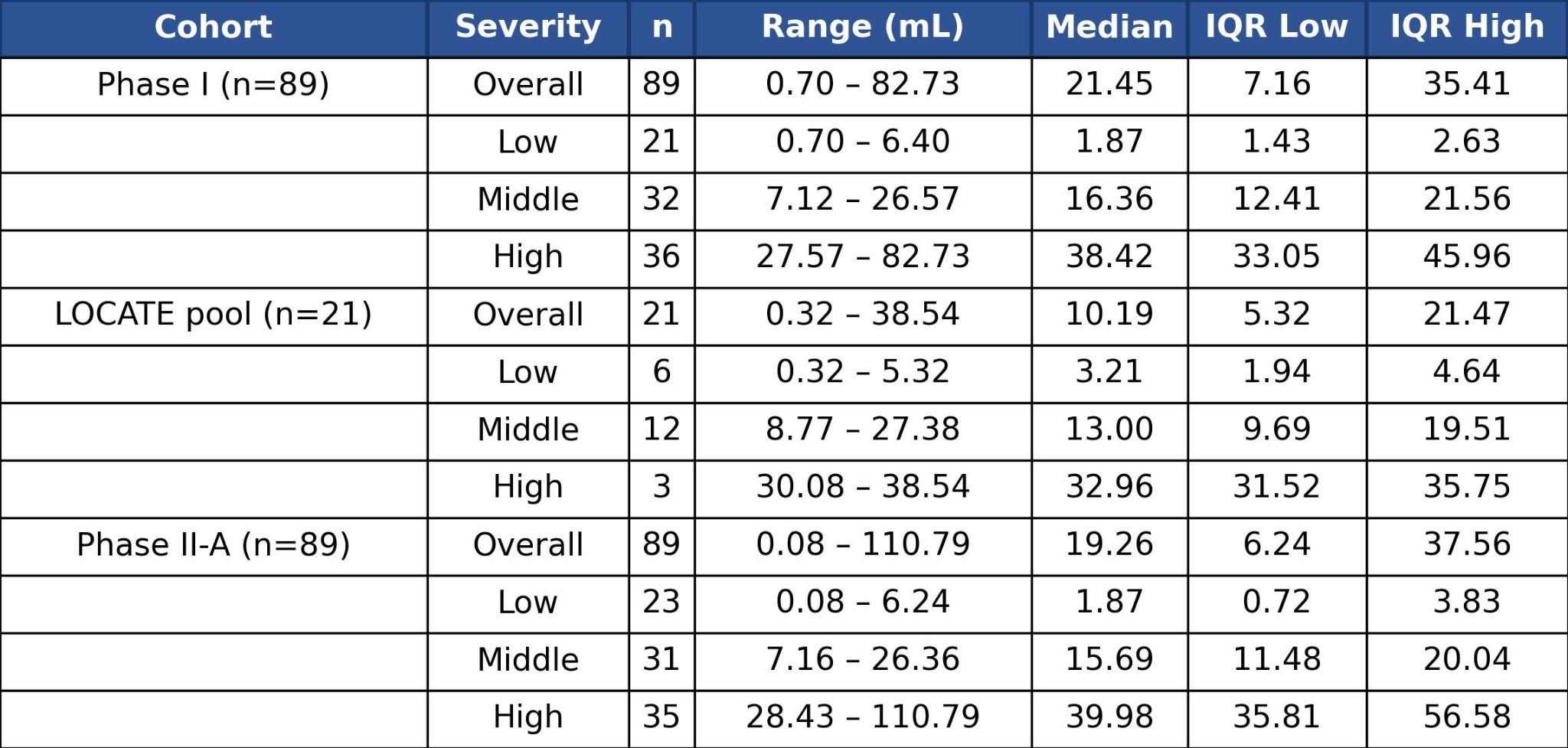


*Severity cutoffs were derived from a Decision Tree classifier trained on the Challenge dataset (n=40): low (≤6.96 mL), middle (6.96–27.40 mL), high (>27.40 mL). ROI_Volume = total white matter hyperintensity volume from manual segmentation; IQR = interquartile range (25th–75th percentile). All values in mL.*

**Supplemental Table S5.** Pairwise comparisons across thresholding strategies (Phase I, LOCATE thresholding, train=inpainted, test=inpainted, n=89).


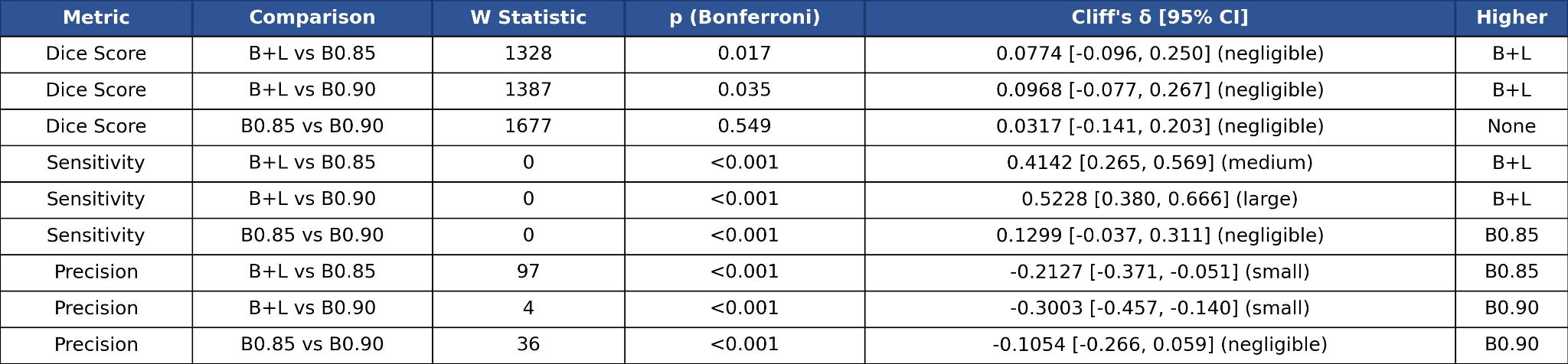


*Pairwise comparisons of Dice score, sensitivity, and precision across three thresholding strategies (B+L, B0.85, B0.90) using Wilcoxon signed-rank tests with Bonferroni correction (k=3 per metric, adjusted α=0.0167). Per-subject metrics represent means across 10 random seeds × 5-fold stratified cross-validation (n=89). Train=inpainted, test=inpainted. Effect size classification follows Hess and Kromrey (2004): negligible (|δ| < 0.147), small (0.147 ≤ |δ| < 0.33), medium (0.33 ≤ |δ| < 0.474), large (|δ| ≥ 0.474). CI=confidence interval.*

***Supplemental Table S6:*** Cluster-level metric comparisons across thresholding strategies (Phase II-A, n=89).


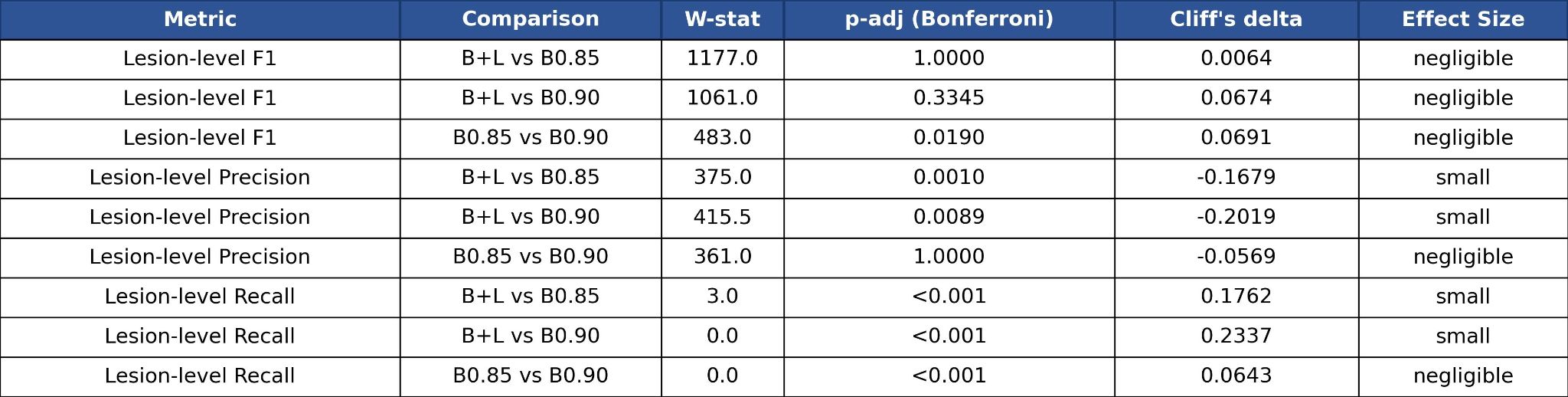


*Wilcoxon signed-rank tests comparing cluster-level F1, precision, and recall between BIANCA threshold configurations (B+L, B0.85, B0.90). Bonferroni correction applied per metric family (k=3 pairwise comparisons, corrected α=0.0167). Cluster-level F1 showed no meaningful differences across thresholding strategies (all Cliff's δ ≤ 0.07, negligible), whereas recall differed substantially, with LOCATE yielding higher recall than both fixed thresholds (Cliff's δ = 0.18–0.23, small). Precision was higher for fixed thresholds relative to LOCATE (Cliff's δ = −0.17 to −0.20, small). Effect sizes classified following Hess and Kromrey (2004): negligible (|δ| < 0.147), small (0.147 ≤ |δ| < 0.474), medium 0.147 ≤ |δ| < 0.474), large (|δ| ≥ 0.474).*

***Supplemental Table S7.*** Pairwise training condition comparisons (Phase I, LOCATE thresholding, test=inpainted, n=89).


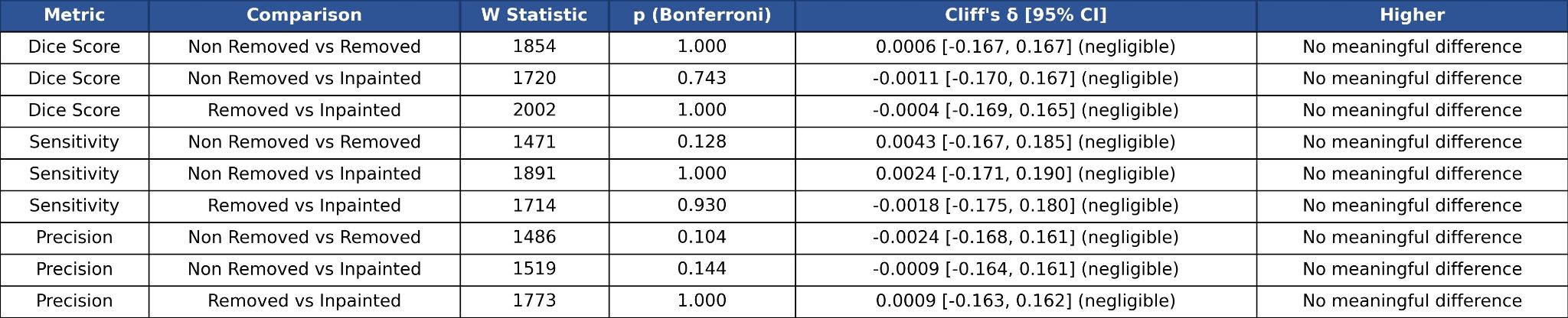


*Bonferroni-corrected pairwise comparisons (k=3 per metric, adjusted α = 0.0167) of Dice score, sensitivity, and precision across Non removed, Removed, and Inpainted training conditions. Per-subject values represent means across 10 seeds × 5-fold cross-validation. All comparisons were non-significant (smallest adjusted p = 0.104), and all Cliff's Delta values were negligible (max |δ| = 0.004). Effect size thresholds follow Hess and Kromrey (2004). CI = confidence interval.*

***Supplemental Table S8.*** Pairwise test condition comparisons (Phase I, LOCATE thresholding, train=inpainted, n=89).


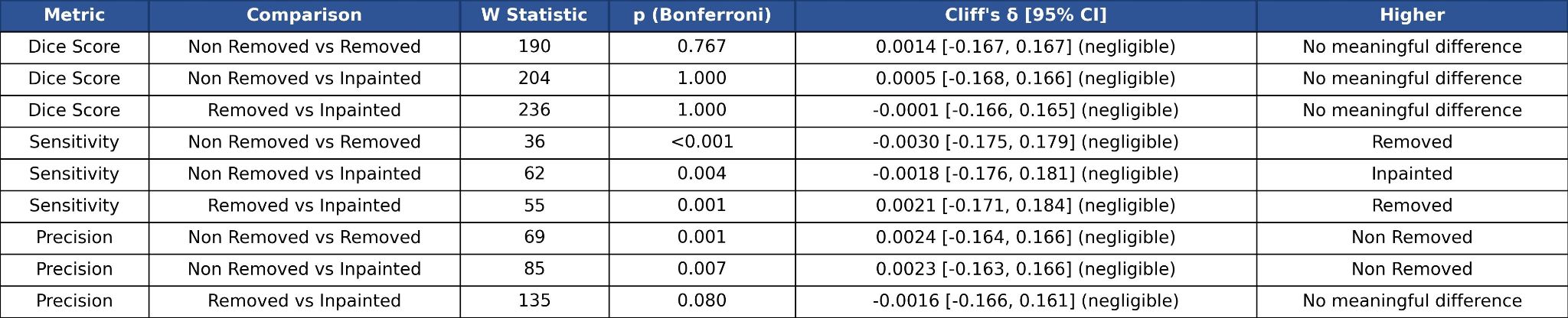


*Bonferroni-corrected pairwise comparisons (k=3 per metric, adjusted α = 0.0167) of Dice score, sensitivity, and precision across Non removed, Removed, and Inpainted test conditions. Cliff's Delta with bootstrapped 95% confidence intervals (1,000 iterations). Effect size thresholds follow Hess and Kromrey (2004). Per-subject values represent means across 10 seeds × 5-fold cross-validation.*

***Supplemental Table S9:*** Comparison of WMH segmentation volumes between non removed and removed conditions stratified by scanner type (Phase II-B, n=211).

***
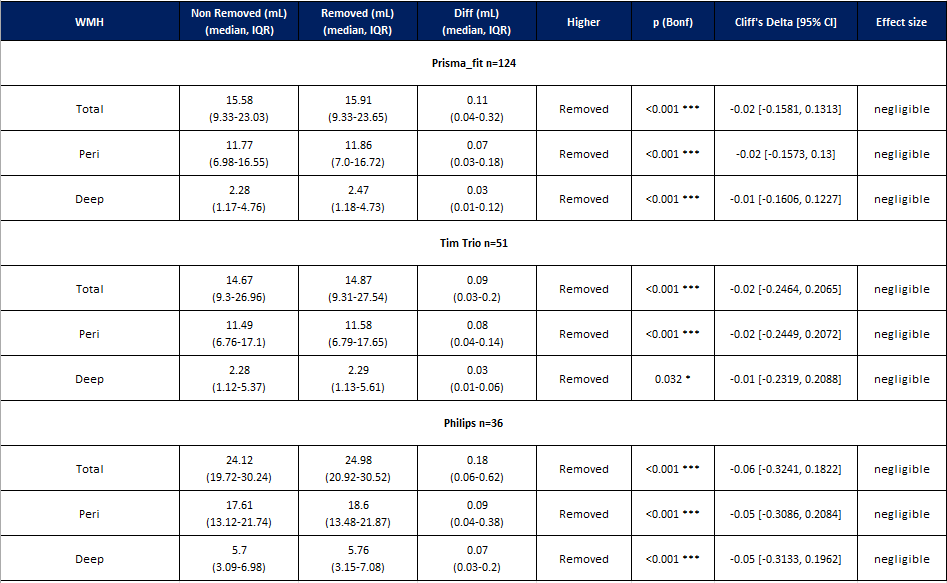
***

*Bonferroni correction applied per family (k=9 tests, 3 scanners × 3 compartments, corrected α=0.0056). Philips (n=36) showed the largest median differences (total 0.18 mL, δ=−0.06) compared to Prisma fit (n=125, total 0.11 mL, δ=−0.02) and Tim Trio (n=51, total 0.09 mL, δ=−0.02), though all effect sizes remained negligible.*

***Supplemental Table S10*** Spearman correlations between stroke lesion volume and WMH volume difference stratified by lesion type (Phase II-B, n=211).


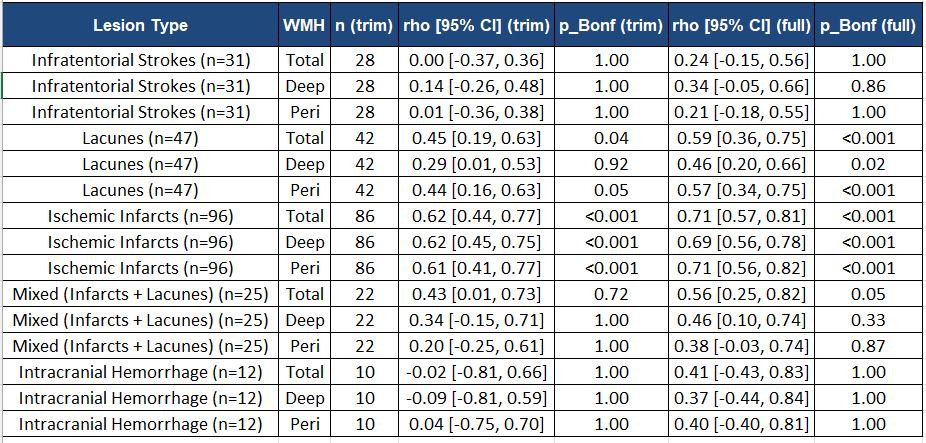


*Correlations between stroke lesion volume and WMH volume difference (Removed minus Non removed) are reported for each lesion type and WMH compartment. Trimmed correlations exclude cases above the 90th percentile of stroke lesion volume per subgroup. Bootstrapped 95% confidence intervals (1,000 iterations). Bonferroni correction applied per family (k=15, five lesion types × three compartments, adjusted α = 0.003). WMH = white matter hyperintensity, Peri = periventricular, rho = Spearman rank correlation coefficient, CI = confidence interval, p_Bonf = Bonferroni-corrected p-value.*

**Supplemental Figure S1:** Threshold optimization analysis across the full probability range (Phase II-A, n=89).

**
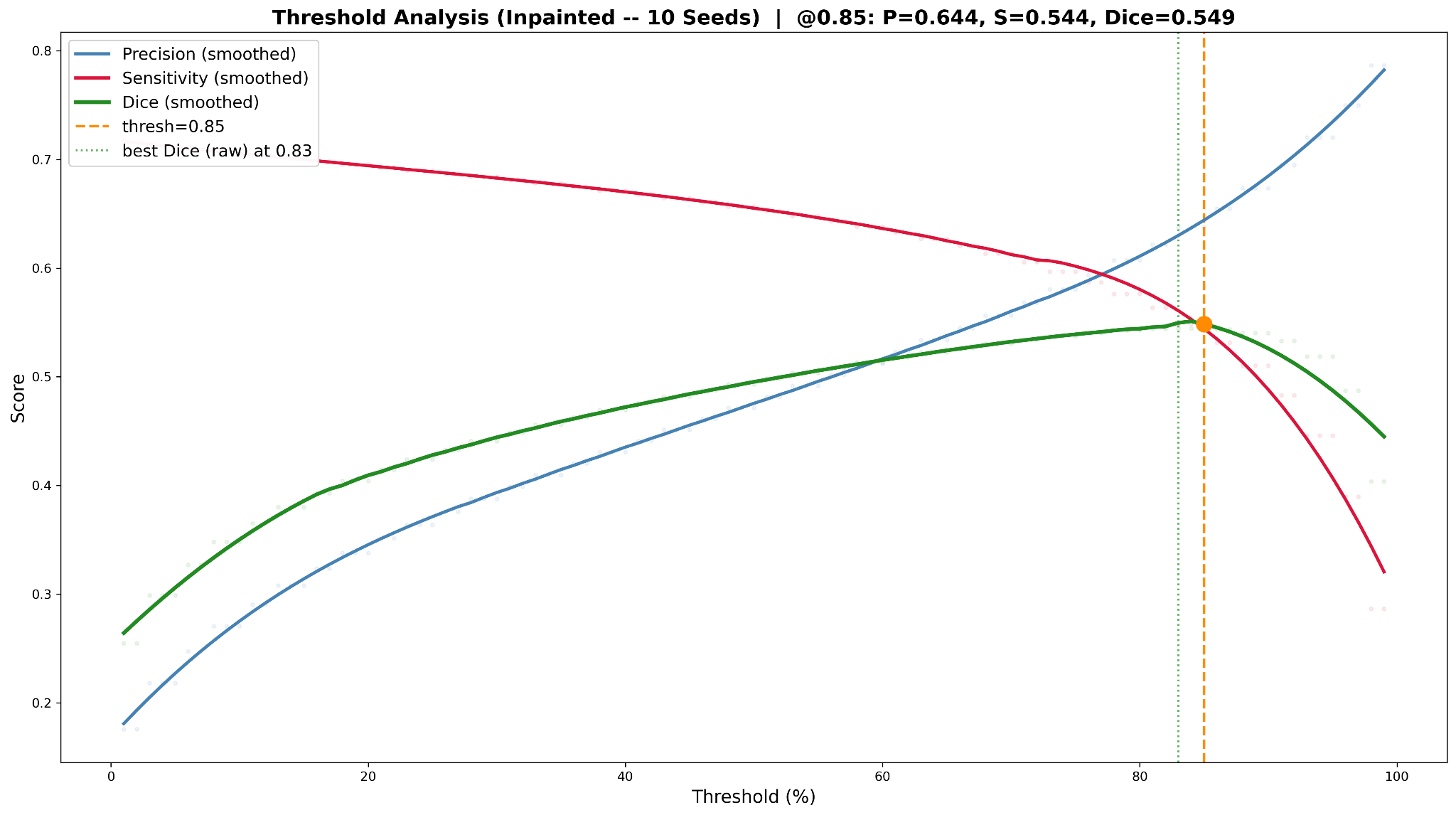
**

*Smoothed performance metrics (Savitzky-Golay filter) plotted against BIANCA probability thresholds (0–100%) for the inpainted condition, averaged across 10 seeds × 5-fold stratified cross-validation. Blue: precision; red: sensitivity; green: Dice coefficient. The orange dashed line marks the selected operating threshold (B0.85); the grey dotted line indicates the empirical Dice maximum at 0.83. At threshold B0.85, the algorithm achieved balanced performance (precision=0.644, sensitivity=0.544, Dice=0.549). The precision-sensitivity crossover near this threshold confirms that B0.85 provides a near-optimal trade-off between false positive control and lesion detection, consistent with prior validation by Ferris et al. (2023). Smoothing was applied for visualization only; all statistical analyses used raw values. Orange marker indicates performance at the selected threshold.*

**Supplemental Figure S2:** Dice score comparison across thresholding strategies by WMH severity (Phase II-A, n=89).**
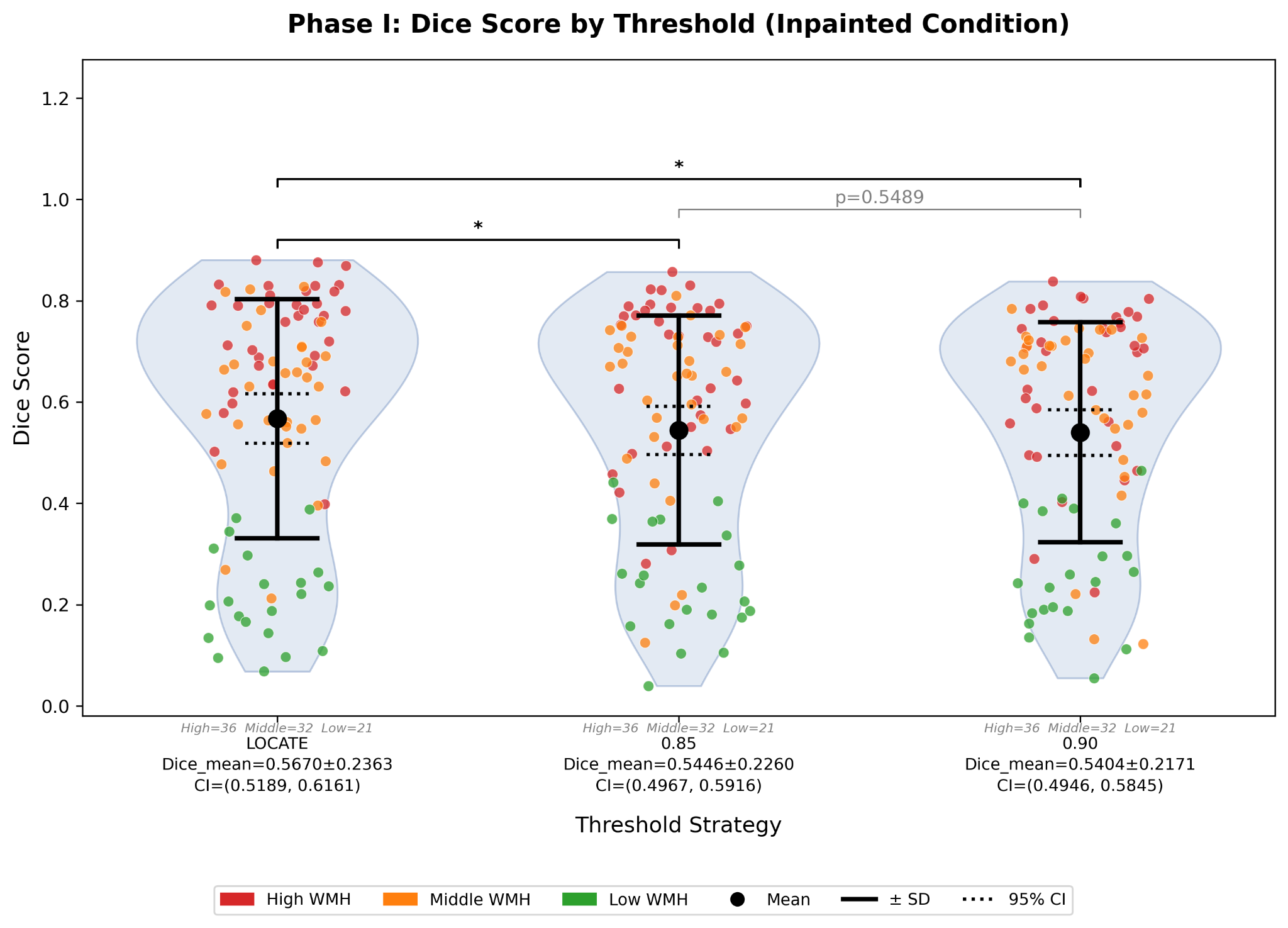
**

*Voxel-level Dice distributions by thresholding strategy (LOCATE [B+L], B0.85, B0.90; train=inpainted, test=inpainted), color-coded by WMH severity tercile (low: <8.78 mL, n=21; middle: 8.78–26.95 mL, n=32; high: >26.95 mL, n=36). Black dots = group means; solid lines = ±1 SD; dotted lines = 95% CI. Brackets indicate Bonferroni-corrected Wilcoxon signed-rank tests (k=3, adjusted α=0.0167). B+L achieved significantly higher Dice than B0.85 and B0.90 (both p<0.017), though effect sizes were negligible (Cliff's δ≤0.10; Supplemental Table S5). Performance scaled with WMH burden (high: 0.60–0.85; low: <0.40).*

**Supplemental Figure S3:** Relationship between voxel-level Dice coefficient and cluster-level F1 score across thresholding strategies (Phase II-A, n=89 ).

**
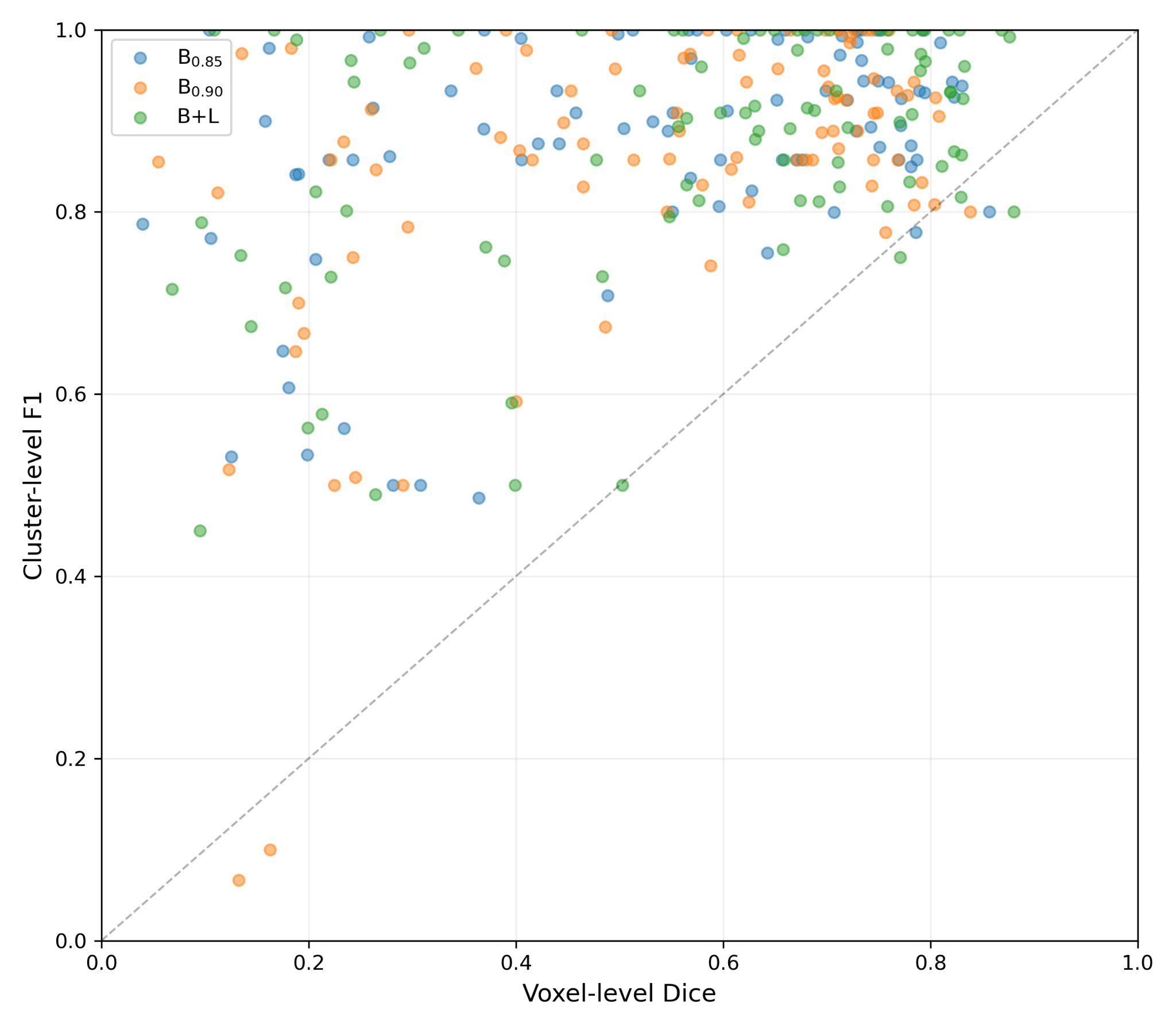
**

*Each point represents one subject under one of three thresholding conditions: B0.85 (blue), B0.90 (orange), and B+L (green). The dashed diagonal indicates the identity line (Dice = Cluster F1). The majority of data points fall above the identity line, indicating that cluster-level F1 consistently exceeded voxel-level Dice, particularly for subjects with low WMH burden (Dice < 0.3), where cluster-level F1 frequently exceeded 0.7. This dissociation demonstrates that BIANCA correctly identifies most WMH clusters even when voxel-level overlap is limited, suggesting that low Dice scores in low-severity cases reflect imprecise cluster boundary delineation rather than failure to detect lesions. Training condition: inpainted; test condition: inpainted. Cluster-level metrics were computed using connected-component labeling (26-connectivity) with empirically determined minimum cluster sizes (see Methods).*

***Supplemental Figure S4:*** Cluster-level F1 comparison across thresholding strategies by WMH severity (Phase II-A, n=89).

*
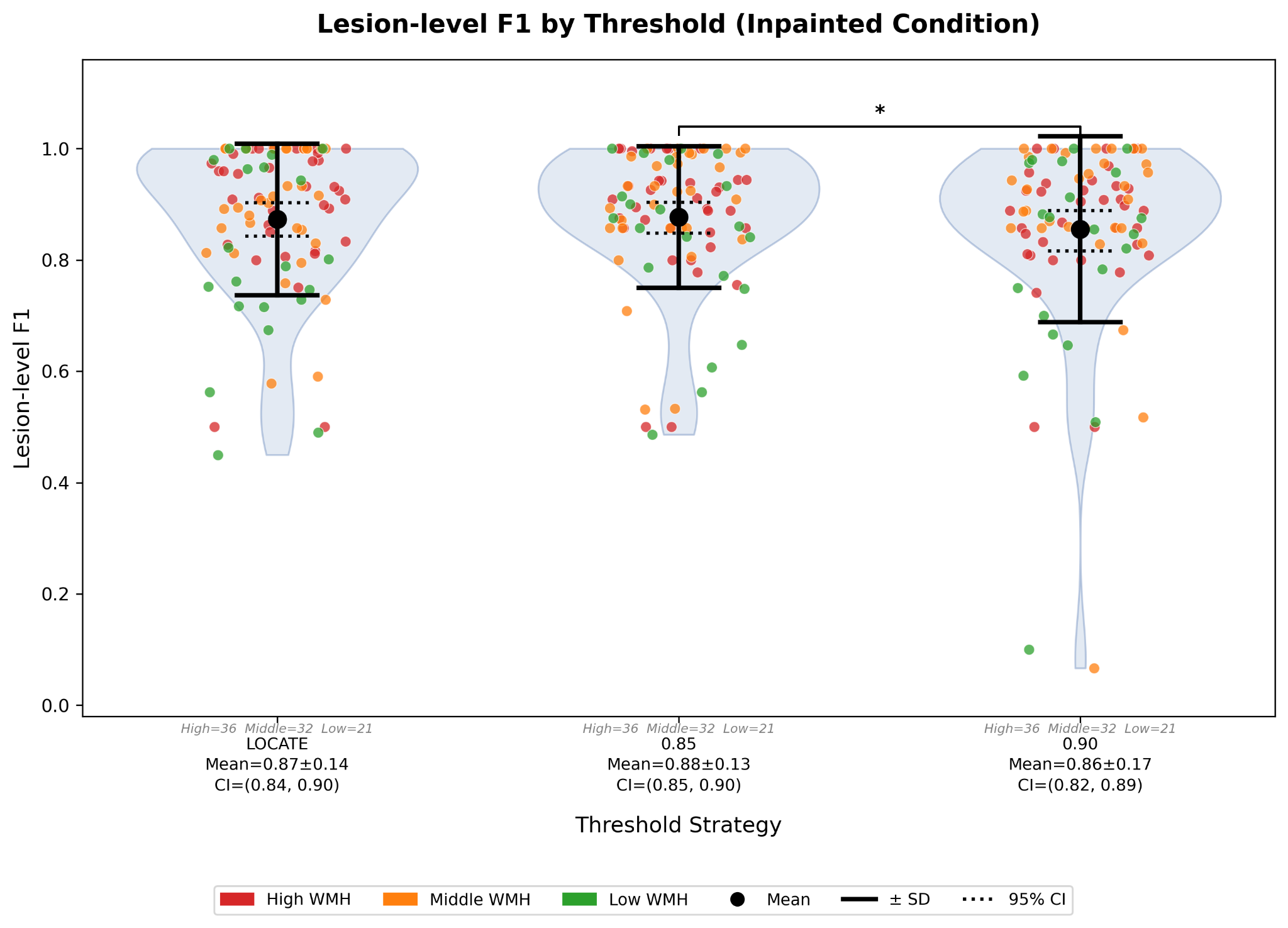
*

*Cluster-level F1 distributions by thresholding strategy (LOCATE [B+L], B0.85, B0.90; train=inpainted, test=inpainted), color-coded by WMH severity tercile (low: <8.78 mL, n=21; middle: 8.78–26.95 mL, n=32; high: >26.95 mL, n=36). Black dots = group means; solid lines = ±1 SD; dotted lines = 95% CI. F1 was uniformly high across strategies (LOCATE: 0.87±0.14; B0.85: 0.88±0.13; B0.90: 0.86±0.17). Only B0.85 vs. B0.90 reached significance after Bonferroni correction (p<0.017); all effect sizes were negligible (Cliff's δ≤0.07; Supplemental Table S6).*

***Supplemental Figure S5.*** Dice similarity coefficients by training condition (Phase I, LOCATE thresholding, test=inpainted, n=89).

*
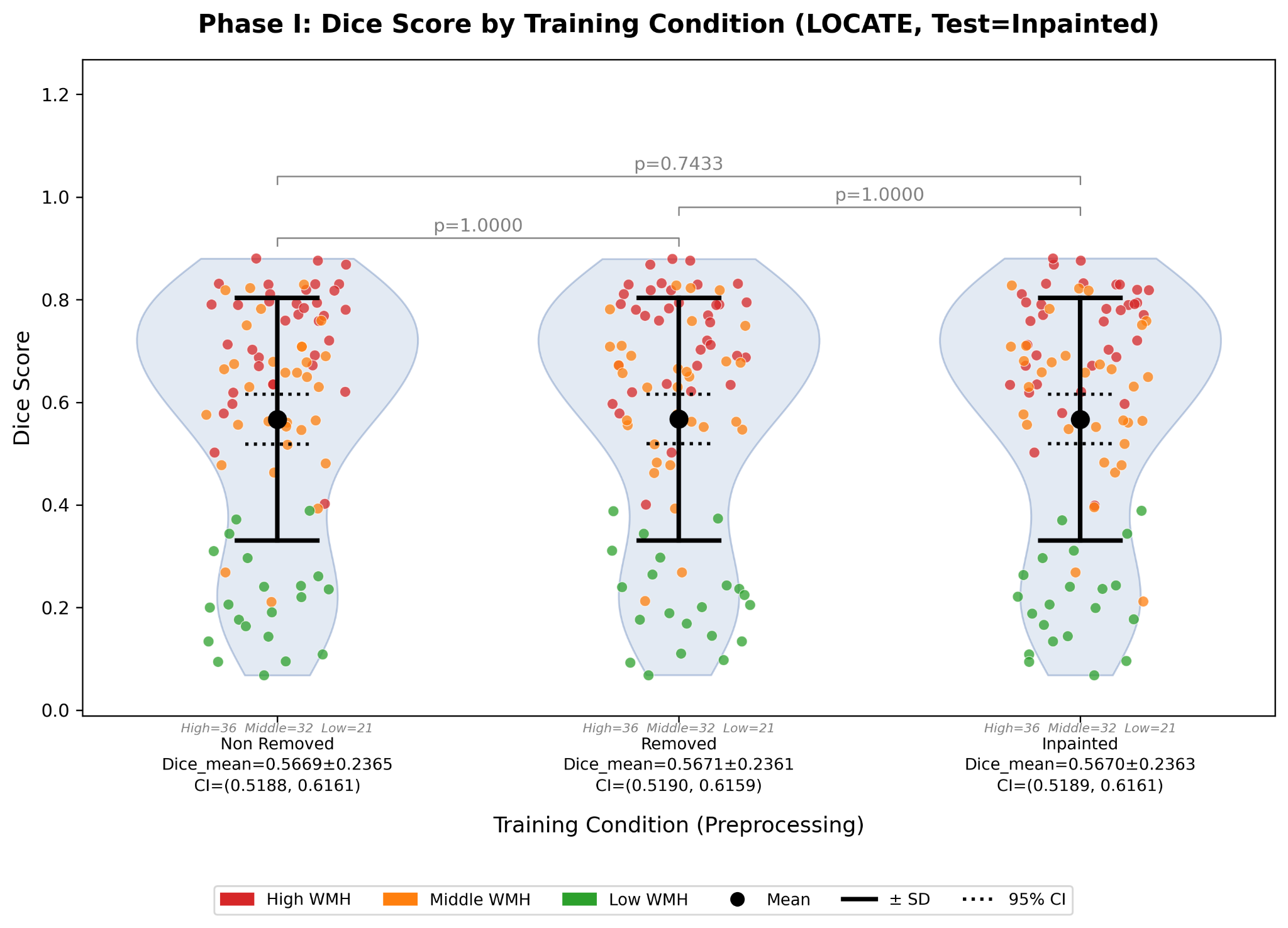
*

*Dice distributions by training condition (non removed, removed, inpainted; LOCATE thresholding, test=inpainted), color-coded by WMH severity tercile (high, n=36; middle, n=32; low, n=21). Black dots = group means; solid lines = ±1 SD; dotted lines = 95% CI. Mean Dice scores were virtually identical across conditions (0.5669–0.5671), with differences confined to the fourth decimal place.*

***Supplemental Figure S6.*** Dice similarity coefficients by test condition (Phase I, LOCATE thresholding, train=inpainted, n=89).

*
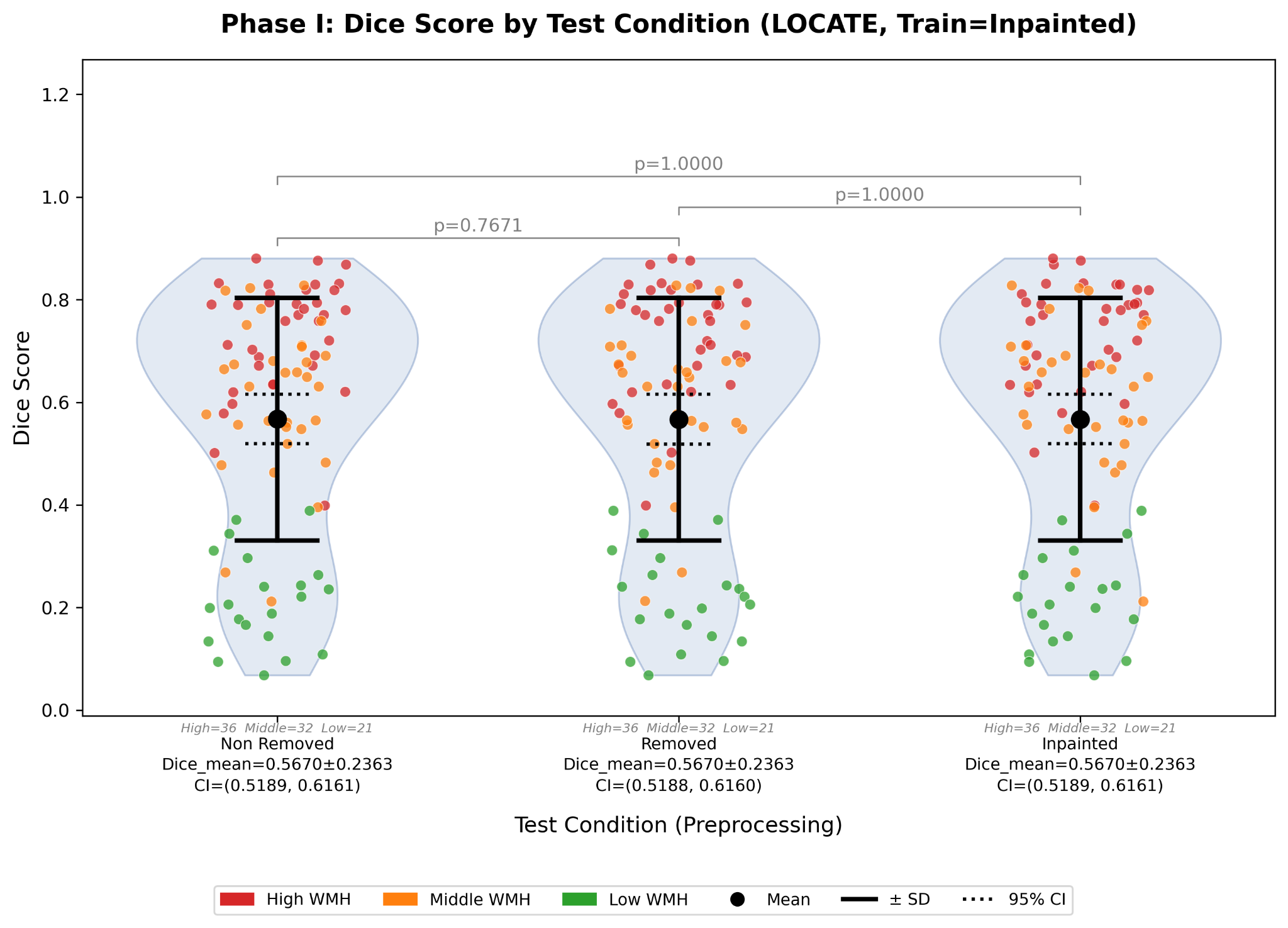
*

*Violin plots show the distribution of Dice scores across non removed, removed, and inpainted test conditions. Individual data points are colored by WMH severity tercile (red=high, orange=middle, green=low; severity group sizes: high n=36, middle n=32, low n=21). Black dots indicate group means, error bars represent ±1 SD, and dotted lines denote 95% confidence intervals. Mean Dice scores were identical across conditions (0.5670±0.2363).*

**Supplemental Figure S7:** SHAP feature importance analysis revealing scanner type as the second most influential factor in the expanded Phase II-B cohort (n=211)


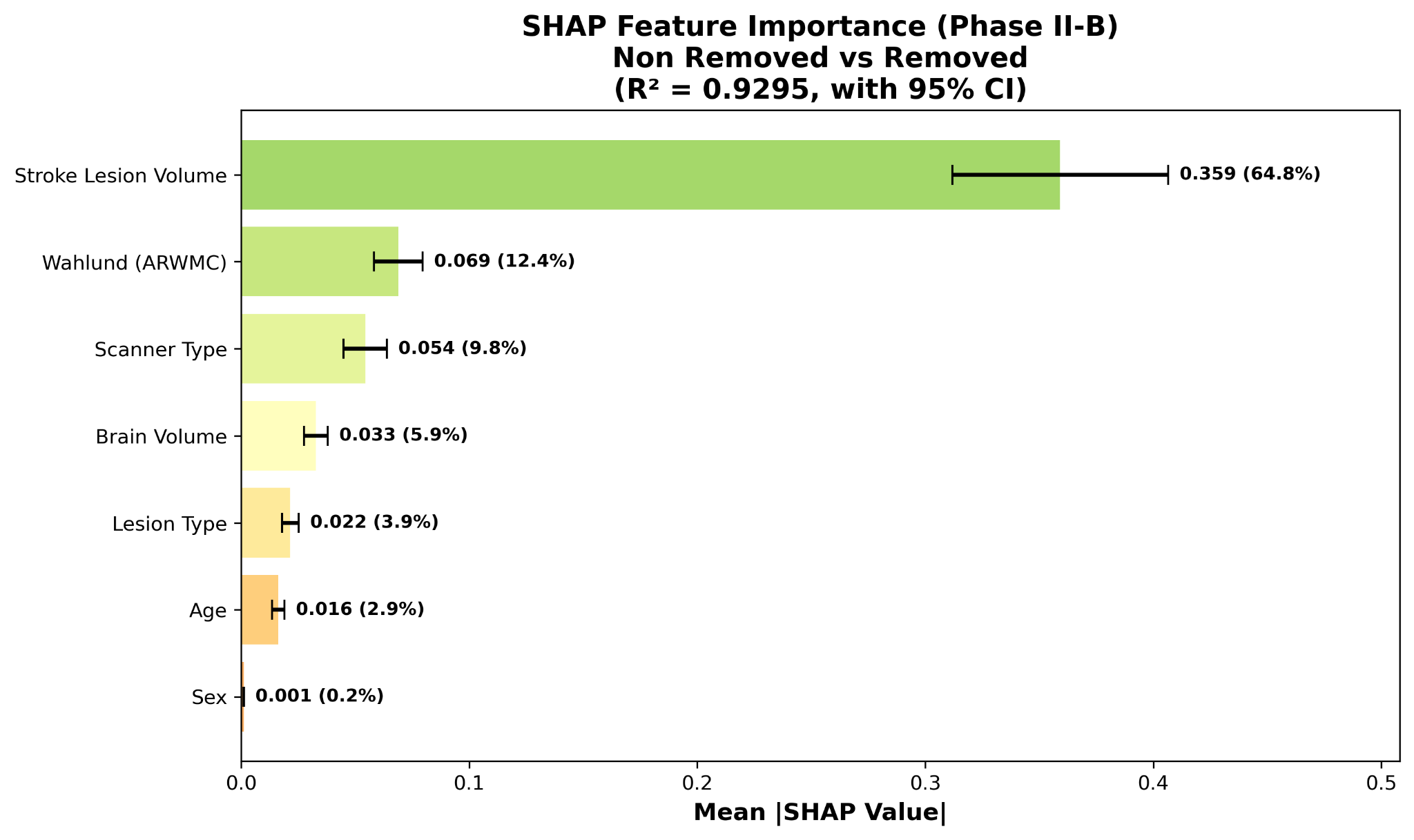


*SHAP feature importance analysis identifies stroke lesion volume as the highest-ranked predictor of WMH volume differences between Non removed and Removed conditions in the expanded Phase II-B cohort (n=211, R² = 0.93). Scanner type ranked third (9.8%), showing a 12-fold increase in relative importance compared to Phase II-A (0.8%), consistent with improved Philips scanner representation. SHAP values represent mean absolute impact on model output; error bars indicate 95% confidence intervals. ARWMC = age-related white matter changes score (Wahlund et al., 2001).*

***Supplemental Figure S8.*** *Effect of stroke lesion removal on FLAIR intensity distributions. (A–C)*

*
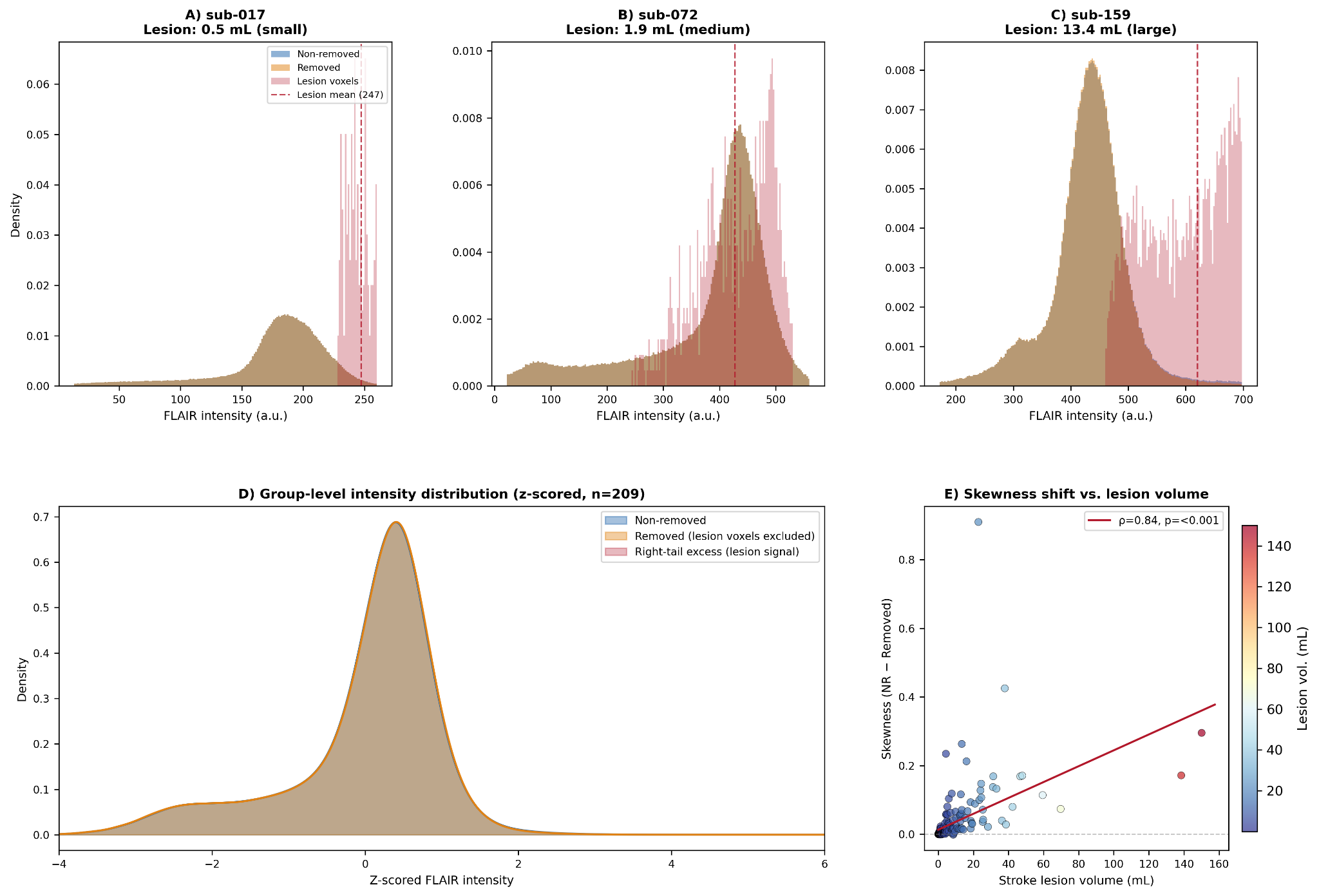
*

*Subject-level intensity histograms for three representative cases with small (0.5 mL), medium (1.9 mL), and large (13.4 mL) stroke lesions, comparing non removed (blue) and removed (orange) conditions. Red bars indicate lesion voxel intensities; dashed red line marks the lesion mean intensity. Lesion removal eliminates the right-tail excess caused by hyperintense stroke voxels, with the effect scaling with lesion size. (D) Group-level z-scored FLAIR intensity distributions (n=209; 2 subjects excluded due to processing failures), showing the non removed (blue) and removed (orange) conditions overlaid. The shaded pink area highlights the right-tail excess attributable to stroke lesion signal. (E) Spearman correlation between stroke lesion volume and skewness shift (non removed minus removed), colored by lesion volume (mL). Larger lesions produced greater skewness reduction upon removal (ρ=0.84, p<0.001), confirming that the distributional distortion scales with lesion burden.*
